# Reinforcement Learning to Prevent Acute Care Events Among Medicaid Populations: Mixed Methods Study

**DOI:** 10.2196/74264

**Published:** 2025-10-08

**Authors:** Sanjay Basu, Bhairavi Muralidharan, Parth Sheth, Dan Wanek, John Morgan, Sadiq Patel

**Affiliations:** 1 Waymark San Francisco, CA United States

**Keywords:** artificial intelligence, AI, clinical decision support, care management, machine learning, reinforcement learning, community health worker, social determinants of health

## Abstract

**Background:**

Multidisciplinary care management teams must rapidly prioritize interventions for patients with complex medical and social needs. Current approaches rely on individual training, judgment, and experience, missing opportunities to learn from longitudinal trajectories and prevent adverse outcomes through recommender systems.

**Objective:**

This study aims to evaluate whether a reinforcement learning approach could outperform standard care management practices in recommending optimal interventions for patients with complex needs.

**Methods:**

Using data from 3175 Medicaid beneficiaries in care management programs across 2 states from 2023 to 2024, we compared alternative approaches for recommending “next best step” interventions: the standard experience-based approach (status quo) and a state-action-reward-state-action (SARSA) reinforcement learning model. We evaluated performance using clinical impact metrics, conducted counterfactual causal inference analyses to estimate reductions in acute care events, assessed fairness across demographic subgroups, and performed qualitative chart reviews where the models differed.

**Results:**

In counterfactual analyses, SARSA-guided care management reduced acute care events by 12 percentage points (95% CI 2.2-21.8 percentage points, a 20.7% relative reduction; *P*=.02) compared to the status quo approach, with a number needed to treat of 8.3 (95% CI 4.6-45.2) to prevent 1 acute event. The approach showed improved fairness across demographic groups, including gender (3.8% vs 5.3% disparity in acute event rates, reduction 1.5%, 95% CI 0.3%-2.7%) and race and ethnicity (5.6% vs 8.9% disparity, reduction 3.3%, 95% CI 1.1%-5.5%). In qualitative reviews, the SARSA model detected and recommended interventions for specific medical-social interactions, such as respiratory issues associated with poor housing quality or food insecurity in individuals with diabetes.

**Conclusions:**

SARSA-guided care management shows potential to reduce acute care use compared to standard practice. The approach demonstrates how reinforcement learning can improve complex decision-making in situations where patients face concurrent clinical and social factors while maintaining safety and fairness across demographic subgroups.

## Introduction

### Background

Health care delivery increasingly relies on multidisciplinary teams, including care coordinators and community health workers, to support patients with complex medical and social needs [1]. These care management programs are now mandated components of state Medicaid programs serving more than 80 million Americans and are widely implemented in Medicare Advantage plans serving an additional 30 million beneficiaries [1]. These programs aim to reduce acute care use through proactive outreach, offering services such as care coordination, medication adherence counseling, and social needs assistance. While physicians and nurses typically follow evidence-based protocols, newer team member disciplines, such as community health workers, frequently lack standardized guidance for prioritizing interventions, instead relying on varied training approaches and personal experiences that may not consistently yield optimal outcomes [2].

This gap is particularly challenging when caring for patients with multiple chronic conditions, mental health disorders, substance use issues, and social needs. In time-constrained interactions, team members must make sequential decisions with long-term consequences, rapidly prioritizing among competing needs while recognizing that today’s choices affect future patient states [3]. Current Medicaid care management programs typically rely on electronic health record documentation and clinical guidelines focused primarily on optimizing each individual’s medical condition, with minimal decision support tools addressing social needs integration or optimal intervention sequencing.

The sequential nature of care management decisions, combined with the need to learn optimal intervention timing from longitudinal patient trajectories, suggests the potential utility of reinforcement learning approaches [4]. Unlike traditional supervised learning methods that treat each decision independently, reinforcement learning explicitly models how current actions influence future states and outcomes [5]. Traditional machine learning approaches cannot effectively capture these temporal dependencies and delayed rewards that characterize care management decisions. While large language models have shown promise in health care, they lack the ability to learn explicit action-value functions from sequential decision processes with delayed rewards [6].

Within reinforcement learning, the state-action-reward-state-action (SARSA) algorithm is particularly well-suited for clinical decision support due to its on-policy learning approach, which ensures that recommendations align with actual practice patterns while still optimizing for improved outcomes [7]. This on-policy characteristic provides crucial safety advantages over off-policy algorithms, such as Q-learning or deep Q-networks, which may recommend actions that deviate substantially from established clinical practice patterns and could potentially compromise patient safety. Unlike off-policy algorithms, SARSA’s conservative on-policy approach provides an important safety constraint for health care applications where drastic deviations from established practice patterns may pose serious health risks.

Currently, care management teams typically rely on training that codifies expert knowledge into predefined curricula on how to address each patient’s needs, often leaving the prioritization of multiple needs to team member experience [8]. This approach can fail to capture the value of experiences for newer team members early in their careers and address how simultaneous comorbid conditions and social-medical interactions can affect real-time decision-making in care management [9].

### This Study

In this study, we report results obtained upon comparing SARSA-guided care management to the status quo approach of human experience–guided decision-making. Using real-world data from multidisciplinary care teams across 2 states, we performed counterfactual causal inference analyses and qualitative reviews to evaluate each approach’s ability to recommend interventions that reduced subsequent acute care use while maintaining fairness across patient subgroups.

## Methods

### Study Design and Data Source

We conducted a comparative effectiveness study using longitudinal cohort data from Medicaid beneficiaries enrolled in care management programs in Virginia and Washington from 2023 to 2024. The programs involved multidisciplinary teams that engaged patients in community settings. Data were extracted from the programs’ health record systems, capturing demographics, health conditions, social determinants, team interventions, and health care use outcomes. Complete code is available at the link included in Multimedia Appendix 1.

The cohort included Medicaid beneficiaries enrolled in care management between January 1, 2023, and December 31, 2024, with follow-up through February 28, 2025. Patient identification was done using a previously validated extreme gradient boosting algorithm considering chronic conditions, previous health care use, and social determinants to identify individuals at high risk for acute care use [10]. The primary outcome was a composite of emergency department visits and hospitalizations.

### Reinforcement Learning Model Development

We estimated the impact of a SARSA reinforcement learning approach to optimize sequential intervention decisions. The SARSA model belongs to the temporal difference learning family and is well suited for health care applications due to its on-policy learning from multistep patient trajectories rather than optimizing for only the next immediate intervention [11].

The state representation comprised features across multiple domains, such as demographics, clinical metrics (chronic conditions and risk factors), social determinants of health, health care use history, and intervention history. The action space consisted of 9 mutually exclusive interventions derived through clinical expert consensus and existing care management protocols, such as substance use support, mental health support, chronic condition management support, food assistance, housing assistance, transportation assistance, utility assistance, childcare assistance, and watchful waiting. Actions are mutually exclusive at each decision point to prevent combinatorial complexity, with the model selecting the single most appropriate intervention based on the current patient state.

For function approximation, we developed a neural network architecture with an input layer accepting the multidimensional state vector, followed by 2 hidden layers with rectified linear unit activation functions and dropout regularization. The output layer contained 9 nodes corresponding to the Q-values for each intervention. Complete architectural specifications, hyperparameter grids, and training procedures are detailed in Multimedia Appendix 1.

### Reward Function Engineering

The reward function captured multiple aspects of clinical quality and safety. The primary component was a negative reward for acute care events, reflecting the main objective of reducing emergency department visits and hospitalizations. We incorporated a continuous reward signal proportional to the reduction in calculated risk score, allowing the model to learn the value of incremental risk reductions even when acute events did not occur.

Clinical appropriateness was encouraged through a structured reward bonus system, including a prevention bonus for avoiding acute care events (scaled based on preintervention risk level) and an intervention matching bonus when the selected intervention aligned with the patient’s primary risk domain. Safety constraints were enforced through both a penalty system and an action masking mechanism that prevented the selection of interventions that violated clinical constraints or were deemed inappropriate based on the patient’s current state.

### Model Training and Evaluation

We divided our dataset into training (2222/3175, 70%), validation (635/3175, 20%), and testing (318/3175, 10%) sets, with stratification by outcome occurrence. All analyses used a fixed random seed (42) to ensure reproducibility. Software versions included Python (version 3.9.7; Python Software Foundation), TensorFlow (version 2.8.0; Google LLC), NumPy (version 1.21.0; NumFOCUS), Pandas (version 1.3.3; NumFOCUS), and Scikit-learn (version 1.0.2; NumFOCUS). The SARSA model was trained for 100 epochs, with early stopping based on validation set performance. Hyperparameters were tuned through grid search on the validation set, optimizing for reduction in acute care events. The code for reproducibility and extension is available at GitHub [12].

### Counterfactual Evaluation

To estimate the clinical impact, we conducted counterfactual causal inference analysis using a held-out test set [13]. For each patient, we generated comprehensive paired trajectories under 2 distinct intervention policies: the status quo approach based on observed care management decisions and the SARSA-guided approach.

Our transition model captured the relationship between patient characteristics, interventions, and outcomes through a neural network architecture trained to predict state evolution conditioned on actions. This model was validated for calibration (expected calibration error of 0.08) and discrimination (area under the curve=0.78).

The transition model architecture comprised an input layer accepting 47 state features; 3 hidden layers with 128, 64, and 32 nodes, respectively; and an output layer predicting next-state probabilities. Additional validation metrics included Brier score (0.142), calibration slope (0.96), and Hosmer-Lemeshow test (*P*=.23), indicating good model fit. We conducted doubly robust sensitivity analysis using inverse probability weighting combined with outcome regression to assess robustness to unmeasured time-varying confounding.

We estimated CIs using bootstrapping with 1000 iterations, resampling at the patient level to account for within-patient correlation in outcomes. This methodology extends traditional causal inference approaches by modeling the complex, sequential nature of care management decisions [14].

We evaluated model performance using multiple metrics, including number needed to treat (NNT), absolute risk reduction (ARR), and relative risk reduction (RRR) with 95% CIs. We assessed fairness across demographic groups by calculating equalized odds discrepancy for self-reported gender, race, and ethnicity. Equalized odds discrepancy is defined as the maximum difference in true positive rates and false positive rates across demographic groups: max(|TPR_group1 – TPR_group2|, |FPR_group1 – FPR_group2|), with values less than or equal to 5% considered acceptable. We selected this metric over demographic parity or calibration measures because it directly assesses whether the model provides equitable benefit (true positive rate) and harm (false positive rate) across groups, which is most clinically relevant for care management decisions [15]. Disparities were assessed on observed outcomes rather than predicted risk scores. Analyses followed the Minimum Information About Clinical Artificial Intelligence Modeling checklist (MI-CLAIM) [16] and Developmental and Exploratory Clinical Investigations of Decision support systems driven by Artificial Intelligence (DECIDE-AI) guidelines [17].

### Qualitative Analysis of Recommendation Differences

We conducted a qualitative analysis of cases where the status quo and SARSA models recommended different interventions, using a grounded theory approach [18]. We selected a purposive sample of 200 patient cases where intervention recommendations differed. To ensure diverse representation, we stratified this sample based on primary risk domain (medical, behavioral, and social) and the presence or absence of acute events following intervention.

Our qualitative analysis followed standard grounded theory methodology with 3 stages of coding [18,19]. Two independent coders with clinical (internal medicine) and social science (medical anthropology and social work) backgrounds conducted the analysis, with regular reflexivity sessions to examine potential bias in interpretation. First, in open coding, we independently reviewed each case, identifying key factors influencing divergent recommendations. Intercoder agreement was assessed using Cohen κ (κ=0.82; indicating substantial agreement). Second, during axial coding, through iterative comparison and refinement, we identified recurring patterns and developed provisional categories describing why and when the models diverged. Finally, in selective coding, we refined these categories into core conceptual themes that explained when the SARSA model’s recommendations differed most substantially from the status quo approach. A complete audit trail documented all coding decisions and category development. We maintained detailed reflexivity notes throughout the process to acknowledge potential researcher bias and ensure analytic rigor.

### Ethical Considerations

This study was approved by the Western Institutional Review Board–Copernicus Institutional Review Board (20243894). All data were deidentified using the safe harbor method to preserve patient privacy, and waiver of consent was obtained from the institutional review board; patients were not compensated for participation.

## Results

### Characteristics of the Study Population

Table 1 presents the demographic and clinical characteristics of the 3175 Medicaid beneficiaries enrolled in care management programs. The study population had a mean age of 34.5 (SD 18.5) years, with 2089 (65.8%) female participants. Most patients were categorized under other or multiple races (2181/3175, 68.7%), with smaller proportions identifying as White (423/3175, 13.3%), Black (376/3175, 11.8%), Hispanic (162/3175, 5.1%), or Asian (33/3175, 1%). The most common chronic conditions were hypertension (1371/3175, 43.2%), depression (1203/3175, 37.9%), and diabetes (939/3175, 29.6%). At baseline, 1642 (51.7%) participants had experienced at least 1 emergency department visit in the previous 6 months, and 738 (23.2%) had been hospitalized.

**Table 1:** Characteristics of the study population (N=3175).

Characteristic			Values
Age (y), mean (SD)			34.5 (18.5)
Female sex, n (%)			2089 (65.8)
**Racial or ethnic group, n (%)**			
	Asian	33 (1)	
	Black	376 (11.8)	
	Hispanic	162 (5.1)	
	White	423 (13.3)	
	Other or multiple races	2181 (68.7)	
**Clinical conditions, n (%)**			
	Hypertension	1371 (43.2)	
	Depression	1203 (37.9)	
	Diabetes	939 (29.6)	
	Substance use disorder	635 (20)	
	Chronic obstructive pulmonary disease	476 (15)	
	Congestive heart failure	349 (11)	
**Social determinants, n (%)**			
	Housing instability	869 (27.4)	
	Food insecurity	730 (23)	
	Transportation barriers	571 (18)	
	Utility needs	428 (13.5)	
**Health care use, n (%)**			
	≥1 emergency department visit in the past 6 months	1642 (51.7)	
	≥1 hospitalization in the past 6 months	738 (23.2)	

### Comparative Effectiveness in Reducing Acute Care Events

In our counterfactual analysis comparing SARSA-guided care management to status quo practice, the SARSA approach achieved an acute event rate of 46% compared to 58% in the status quo condition, representing an absolute reduction of 12 percentage points (95% CI 2.2-21.8 percentage points; *P*=.02) and a relative reduction of 20.7% (Table 2). This translates to an NNT of 8.3 (95% CI 4.6-45.2), indicating that for approximately every 8 patients receiving SARSA-guided interventions instead of standard care, 1 acute care event would be prevented. The number needed to harm was infinite, indicating no cases of worsened outcomes because of following SARSA recommendations rather than the status quo approach.

**Table 2:** Effectiveness of the state-action-reward-state-action (SARSA)–guided care management compared to the status quo approach.

Outcome			SARSA, %		Status quo, %		Difference (95% CI)		*P* value
**Primary outcome**									
	Acute care event rate	46		58		–12.0 (–21.8 to –2.2)		.02	
**Secondary outcomes**									
	Absolute risk reduction, percentage points	—^a^		—		12.0 (2.2 to 21.8)		.02	
	Relative risk reduction	—		—		20.7 (3.8 to 37.6)		—	
	Number needed to treat^b^	—		—		8.3 (4.6 to 45.2)		—	
	Number needed to harm	—		—		Not defined (infinite)		—	
**Risk-stratified analysis: number needed to treat** ^b^									
	High-risk patients	—		—		5.2 (3.1 to 14.3)		.004	
	Medium-risk patients	—		—		8.9 (4.7 to 38.6)		.02	
	Low-risk patients	—		—		23.4 (11.2 to infinite)		.08	
**Fairness metrics: equalized odds discrepancy** ^c^									
	Gender	3.8		5.3		–1.5% (0.3% to 2.7%)		.04	
	Race and ethnicity	5.6		8.9		–3.3% (1.1%-5.5%)		.008	

^a^Not applicable.

^b^The number needed to treat is the number of patients who would need to receive SARSA-guided interventions instead of status quo care to prevent 1 acute care event.

^c^Lower equalized odds discrepancy values indicate more equitable performance across demographic groups. The improvement in fairness metrics for the SARSA model compared to the status quo approach represents a 28.3% reduction in gender-based disparities and a 37.1% reduction in race- and ethnicity-based disparities.

The effectiveness of the SARSA-guided interventions varied by patient risk level. We observed the greatest benefit among high-risk patients (top tertile of preintervention risk score), with an NNT of 5.2 (95% CI 3.1-14.3). Medium-risk patients (second tertile) showed moderate benefit with an NNT of 8.9 (95% CI 4.7-38.6), while low-risk patients (bottom tertile) demonstrated less substantial improvement with an NNT of 23.4 (95% CI 11.2-∞).

Sensitivity analysis using doubly robust estimation confirmed the robustness of our primary findings. The doubly robust estimator yielded an ARR of 11.2 percentage points (95% CI 1.8-20.6 percentage points), closely aligning with our primary counterfactual analysis. This consistency suggested that unmeasured time-varying confounding is unlikely to substantially bias our estimates.

### Patterns of Intervention Recommendations

Analysis of the intervention patterns revealed substantial differences between the SARSA approach and status quo practice. The SARSA model recommended substantially more chronic condition management interventions (54% vs 36%, difference +18%) and substance use support (26% vs <0.1%, difference +26%). Conversely, status quo practice featured more mental health support interventions (34% vs 4%, difference –30%), housing assistance (10% vs 3%, difference –7%), and food assistance (9% vs 1%, difference –8%).

The differences in recommendations reflected the algorithm’s learned patterns rather than a devaluation of mental health interventions. When examining cases where the status quo approach recommended mental health support but the SARSA model recommended substance use support, our qualitative analysis found that the SARSA model frequently identified underlying substance use issues contributing to mental health symptoms. The status quo approach often defaulted to treating mental health symptoms (anxiety and depression) without addressing potential substance use triggers.

### Fairness Analysis Across Demographic Groups

The SARSA approach demonstrated improved equalized odds fairness metrics across demographic subgroups compared to status quo approaches (Table 2). For gender, the SARSA model showed a 3.8% disparity in outcomes versus 5.3% for the status quo approach, representing a 28.3% improvement in fairness. For race and ethnicity, the SARSA model demonstrated a 5.6% disparity compared to 8.9% for the status quo approach—a 37.1% improvement.

### Qualitative Findings From Chart Reviews

The qualitative analysis revealed 7 key areas where the SARSA model’s recommendations better recognized complex interactions among medical, behavioral, and social needs (Textbox 1).

**Seven key areas where recommendations by the state-action-reward-state-action (SARSA) model better recognized complex interactions among medical, behavioral, and social needs.**

1. Housing quality and respiratory health interactions: the SARSA model identified cases where poor housing conditions exacerbated respiratory conditions and recommended housing and chronic condition interventions concurrently, whereas these interventions fell further down the list of priorities in the status quo.

2. Food security and chronic disease management: while the status quo approach often addressed either food insecurity or chronic disease separately, the SARSA model linked these needs, recommending food support and chronic condition interventions sequentially when people had these risk factors concurrently.

3. Substance use recognition: the SARSA model more frequently recognized underlying substance use issues in patients presenting with symptoms of anxiety or depression. Rather than solely recommending mental health support (as seen in the status quo approach), the SARSA model appropriately recommended substance use interventions when evidence suggested this was a primary driver of symptoms.

4. Housing stability and mental health: the SARSA model identified connections between housing instability and mental health deterioration, recommending coordinated interventions that addressed both needs, often addressing housing interventions before mental health or alternating between housing and mental health rather than focusing on mental health alone.

5. Transportation and health care access: the SARSA model recognized when transportation barriers directly affected health care access and treatment adherence, prioritizing transportation assistance at critical junctures in care, such as before or after outpatient visits; in the chart review of the status quo approach, transportation was usually addressed only after hospitalizations rather than proactively.

6. Utility access and health-dependent technologies: the SARSA model identified cases where utility issues (eg, electricity and water) affected health-critical technologies (eg, oxygen concentrators and medication refrigeration), whereas the status quo approach often missed these connections and did not offer utility support proactively when patients had durable medical equipment.

7. Social isolation and treatment adherence: the SARSA model recognized patterns where social isolation contributed to poor medication adherence or appointment attendance, recommending interventions that addressed underlying social needs, whereas the status quo approach often leaned toward “watchful waiting” for such patients.

Analysis of the relationship between emergent qualitative themes and quantitative outcomes revealed that cases involving medical-social interaction themes (theme 3: “integrated medical-social needs assessment” and theme 5: “housing-health interactions”) accounted for 65% of the observed ARR. Specifically, patients for whom the SARSA model identified housing and respiratory interactions or food insecurity and diabetes management needs showed the greatest benefit from SARSA-guided interventions compared to status quo care (NNT=4.2 vs overall NNT=8.3).

## Discussion

### Principal Findings

In this study, we examined whether reinforcement learning, specifically a SARSA approach, could effectively assist care management team members in prioritizing interventions for patients with complex medical and social needs. To the best of our knowledge, this is the first application of reinforcement learning beyond physician- or nurse-led medical decisions, entering into multidisciplinary care-team decision-making that now reaches more than 80 million Americans [1]. The SARSA-guided approach reduced acute care events by 12 percentage points compared to status quo practice, with improved fairness metrics across demographic groups. This ARR of 12% and RRR of 20.7% compares favorably to many established clinical interventions. For context, statins for patients with hypertension and cardiovascular risk factors show an ARR of 28% to 54% and RRR of 36% to 67% [20]. Our NNT of 8.3 indicates substantial clinical relevance, falling within the range considered highly effective for many pharmacological and behavioral interventions in chronic disease management [20].

In our qualitative assessment, the greater effectiveness of the SARSA model than the status quo approach appeared to derive from its ability to learn optimal intervention sequences from longitudinal patient trajectories [4,5] rather than relying on individual experience and judgment, which may vary among team members. The algorithm was particularly effective for high-risk patients (NNT=5.2), suggesting its utility in targeting intensive support to those most likely to benefit.

Our mixed methods approach [18] revealed several key features of the SARSA model’s differences from the status quo approach. The qualitative analysis identified 7 distinct patterns where the SARSA model recognized complex interactions between medical and social needs often missed in standard practice [9]. A particular strength of our study was the use of real-world care management data, capturing both medical and social care details often missing from conventional health records [1]. Previous work in health care artificial intelligence (AI) has largely relied on hospital or clinic-based data [21-23], limiting its applicability to community-based care settings where most of the care management occurs [1,8]. Our dataset uniquely captured the full scope of care manager activities, including home visits, social service visits, and “street medicine” interactions.

These findings compare favorably to other care management interventions reported in the literature. Kangovi et al [24] reported an NNT of 6 for preventing one 30-day readmission with community health worker interventions, while Krieger et al [25] found an NNT of 15 for increasing symptom-free days by 1 day per 2 weeks in children with asthma. Our observed NNT of 8.3 suggests that SARSA-guided care management provides efficient targeting of interventions within the range of established care management approaches.

Real-world deployment of SARSA-guided care management faces several implementation challenges beyond technical performance. Workflow integration requires careful consideration of existing care team processes, with recommendations delivered through user interfaces that complement rather than disrupt established clinical workflows. Team training must address both technical literacy and trust building, as care team members need to understand when and why to follow or override algorithmic recommendations. Override mechanisms must be easily accessible and well documented to maintain clinical autonomy while capturing data on recommendation acceptance patterns. Continuous monitoring protocols are essential to detect model drift, reward hacking behaviors, and changes in patient population characteristics that might affect model performance.

The improved fairness metrics achieved by the SARSA model are particularly noteworthy given concerns about AI perpetuating health care disparities [15]. The reduction in outcome disparities across gender (3.8% vs 5.3%) and race and ethnicity (5.6% vs 8.9%) suggests that appropriately designed AI systems may help promote more equitable care delivery. We attribute this improvement to several factors, including the diverse training dataset, explicit consideration of social determinants in the state representation, and the action masking mechanism that prevented unsafe or inappropriate interventions.

While our fairness analysis demonstrates improved equity compared to status quo practice, the risk of unintended bias reinforcement through AI systems requires ongoing vigilance. We recommend implementing comprehensive bias mitigation strategies, including regular algorithmic auditing across demographic subgroups, continuous monitoring of recommendation patterns for evidence of discriminatory outcomes, and establishment of governance frameworks for detecting and addressing potential bias reinforcement. Future monitoring plans should include quarterly fairness assessments, annual model retraining with updated data, and systematic review of override patterns to identify the potential sources of algorithmic bias. In addition, diverse stakeholder engagement in model development and deployment can help identify blind spots that technical metrics alone might miss.

### Limitations

Several limitations should be noted. First, while our counterfactual analyses showed promising results, prospective validation through a randomized trial would provide stronger evidence of effectiveness, particularly as our approach may not fully account for time-varying unmeasured confounders unaddressed by our individual-level causal inference methodology. Second, our training data came from Medicaid populations in 2 states, potentially limiting generalizability to populations with higher incomes and different health care insurance coverage or in regions with different care delivery systems or social service landscapes. Third, while we demonstrated improved fairness metrics [15-17], a longer-term study would be needed to confirm sustained reductions in health care disparities.

The generalizability of our findings is limited by the single-organization implementation across Virginia and Washington state Medicaid programs. Variations in state Medicaid waiver designs, social service infrastructure availability, and electronic health record integration capabilities may substantially affect both state vector quality and action feasibility in other contexts. For example, states with more robust housing assistance programs may see different patterns of housing-related intervention effectiveness, while states with limited mental health infrastructure may require modified action spaces. Successful adaptation to different health care contexts will likely require retraining models with local data, adjusting action spaces to reflect available services, and validating performance across diverse patient populations and care delivery systems.

Several additional limitations warrant consideration. The potential for gaming behaviors (influencing the model to achieve a desired recommendation) exists if care team members learn to manipulate model inputs to achieve preferred recommendations, which could undermine the integrity of the decision support system. We recommend implementing randomized auditing procedures and behavioral monitoring to detect and prevent such gaming. The single-vendor, 2-state implementation limits generalizability to other care management programs with different patient populations, service availability, or organizational structures. Future research should validate these findings across diverse health care contexts and patient populations.

The counterfactual evaluation approach, while methodologically rigorous, relies on assumptions about unmeasured confounding that cannot be fully validated without prospective implementation. Although our sensitivity analyses suggest robustness to unmeasured confounding, prospective randomized trials remain the gold standard for causal inference in clinical interventions. In addition, the 12-month follow-up period may not capture longer-term effects of intervention sequencing decisions, and the focus on acute care use may miss other important outcomes, such as patient satisfaction, quality of life, or care team efficiency.

### Future Directions

Future research should explore several key directions. First, extending the SARSA approach to include more granular social determinants data and community resource availability could further improve intervention targeting. Second, investigating how to optimally integrate AI recommendations into care team workflows while preserving human judgment and relationship-based care delivery will be important. Third, examining how SARSA-guided care management performs across different health care contexts and patient populations could identify areas for refined algorithm development.

Successful integration of SARSA-guided recommendations into care team workflows requires careful attention to user interface design, governance structures, and quality assurance mechanisms. User interfaces should present recommendations with clear rationale, confidence indicators, and easy override options to maintain clinical decision-making autonomy. Governance frameworks must address algorithm change management, including procedures for model updates, performance monitoring, and stakeholder communication. Quality assurance protocols should include regular validation of model predictions against observed outcomes, monitoring for reward hacking behaviors where care teams might game the system, and assessment of recommendation acceptance rates across different team members and patient populations. In addition, clear escalation procedures must be established for cases in which algorithmic recommendations conflict with clinical judgment or patient preferences.

### Conclusions

Our findings suggest that reinforcement learning may effectively support complex care management decisions while promoting more equitable care delivery. As care management programs are mandated benefits for more than 80 million Americans and health care systems increasingly diversify the types of professionals working on these teams to address both medical and social needs, particularly through nonphysician team members for whom standardized guidelines are not universally available, AI approaches that can learn optimal intervention sequences while maintaining safety and fairness will become increasingly valuable. Further research should focus on prospective validation and optimal integration into care team workflows.

## Appendix

Multimedia Appendix 1 Complete model architecture, hyperparameter tuning grids, and code for reproducibility.

## Abbreviations

AI: artificial intelligence
ARR: absolute risk reduction
NNT: number needed to treat
RRR: relative risk reduction
SARSA: state-action-reward-state-action
