# Supplementary material for: Reinforcement Learning to Prevent Acute Care Events Among Medicaid Populations: Mixed Methods Study
Source: JMIR AI. 2025 Oct 8;4:e74264. doi: 10.2196/74264 (PMC12547335; doi:10.2196/74264)
Supplement: Multimedia Appendix 1 [file ai_v4i1e74264_app1.docx]

Supplementary Materials

# Data and Interventions

The study utilized data from the patient relationship manager/health records of a multidisciplinary care team program in two states to create a comprehensive view of patient health status and healthcare utilization. The detailed day-to-day workflows and record systems of the teams were described in a previous clinical publication [1]. The study cohort consisted of unique Medicaid patients who were enrolled in the care team program in targeted geographies (Seattle, WA; Richmond and Hampton Roads, VA) during the implementation period. Patients were identified using a previously published XGBoost machine learning ensemble designed to predict the likelihood of ED visits and hospitalizations.

Intervention Team Composition:

The multidisciplinary intervention team comprised:

- Community Health Workers (CHWs)

- Care Coordinators

- Licensed Social Worker Therapists

- Advanced Practice Pharmacists/Pharmacy Technicians

- Supervising Primary Care Physicians (for triage of clinical concerns)

## Detailed Description of Interventions

1. Substance Use Support:

The care management team provided counseling, referrals, and support for patients with substance use disorders. This included motivational interviewing techniques, connecting patients to local support groups, and coordinating with addiction specialists. For example, team members might accompany patients to their first Narcotics Anonymous meeting or help them navigate the intake process for a medication-assisted treatment program for opioid use disorder. Additionally, team members were trained in SBIRT: screening, brief intervention (typically motivational interviewing), and referral to treatment. Studies have shown that these multidisciplinary interventions can significantly improve engagement in substance use treatment and reduce relapse rates [2].

2. Mental Health Support:

The team offered cognitive behavioral therapy, supported by psychiatry e-Consult services for pharmacotherapy recommendations. Care managers played a crucial role in mental health support by providing regular check-ins, teaching coping strategies, and facilitating connections to mental health professionals. For instance, a team member might help a patient practice mindfulness techniques for anxiety management or assist in setting up and attending telepsychiatry appointments. Research has demonstrated that care management-led mental health interventions can improve symptoms and quality of life for patients with depression and anxiety [3].

3. Chronic Condition Management:

Chronic condition support was further divided by primary disease type for which care managers were trained (asthma/COPD, diabetes, heart failure, hypertension), and intervention type (medication adherence support, appointment support, equipment support, following provider instructions, nutrition planning, or physical activity planning). For example, within hypertension support, care managers working on medication adherence could assist with creating pill organizers, setting up medication reminders, or teaching patients how to use home health monitoring devices like blood pressure cuffs. Team members also helped translate complex medical instructions into actionable steps for patients. Studies have shown that multidisciplinary interventions in chronic disease management can lead to improved health outcomes and reduced hospitalizations [4].

4. Food Assistance:

The team connected patients to food resources and assistance programs, addressing a critical social determinant of health. Care managers helped patients apply for Supplemental Nutrition Assistance Program (SNAP) benefits, located nearby food banks, and even assisted with meal planning and grocery shopping for those with dietary restrictions related to their health conditions. Research has shown that interventions addressing food insecurity can lead to improved dietary habits and better management of chronic conditions like diabetes [5].

5. Housing Assistance:

The care management team supported patients in finding stable housing and navigating housing programs. This included helping patients complete housing applications, accompanying them to housing authority appointments, and connecting them with local shelters or transitional housing options when necessary. For patients at risk of eviction, team members might help negotiate with landlords or connect patients to legal aid services. Studies have demonstrated that housing stability interventions can reduce emergency department visits and improve overall health outcomes [6].

6. Transportation Assistance:

The team arranged transportation to medical appointments and social services, addressing a common barrier to care. This might involve helping patients navigate public transportation systems, coordinating rides with volunteer driver programs, or assisting in applying for medical transportation benefits. Care managers also educated patients on how to schedule and use para-transit services. Research has shown that addressing transportation barriers through multidisciplinary interventions can significantly improve appointment adherence and reduce missed care opportunities [7].

7. Utility Assistance:

Care managers helped patients access utility assistance programs, ensuring they maintained essential services like electricity, water, and heating. This could involve helping patients negotiate payment plans with utility companies, apply for energy assistance programs, or connect with local charities that provide emergency utility payment assistance. Addressing utility insecurity has been shown to reduce stress and improve overall health outcomes, particularly for patients with chronic conditions that require electricity-dependent medical equipment [8].

8. Child Care Assistance:

The team supported patients in finding and accessing childcare services, recognizing this as a critical factor in enabling patients to attend medical appointments and engage in their own healthcare. Care managers might help patients apply for childcare subsidies, identify local daycare centers or after-school programs, or coordinate with family members to ensure child care coverage during medical appointments. Studies have shown that addressing childcare needs can improve healthcare utilization and employment outcomes for low-income parents [9].

9. No Intervention (‘Watchful Waiting’, or ‘Continue Current Care’):

In cases where patients were stable and additional interventions were not immediately necessary, the team maintained periodic check-ins to monitor the patient's status and ensure they remained connected to their primary care provider. This approach aligns with the principle of providing the right level of care at the right time, preventing unnecessary interventions while maintaining a safety net for patients [10].

Bibliography:

1. Baum A, Batniji R, Ratcliffe H, DeGosztonyi M, Basu S. Supporting Rising-Risk Medicaid Patients Through Early Intervention. NEJM Catal Innov Care Deliv. 2024;5(11). DOI: 10.1056/CAT.24.0060.

2. Samet JH, Blokhina E, Cheng DM, et al. A Strengths-Based Case Management Intervention to Link HIV-Positive People Who Inject Drugs in Russia to HIV Care. AIDS. 2019;33(9):1467-1476.

3. Coventry PA, Hudson JL, Kontopantelis E, et al. Characteristics of Effective Collaborative Care for Treatment of Depression: A Systematic Review and Meta-Regression of 74 Randomised Controlled Trials. PLoS One. 2014;9(9):e108114.

4. Joo JY, Liu MF. Experiences of Case Management with Chronic Illnesses: A Qualitative Systematic Review. Int Nurs Rev. 2019;66(1):102-113.

5. Seligman HK, Lyles C, Marshall MB, et al. A Pilot Food Bank Intervention Featuring Diabetes-Appropriate Food Improved Glycemic Control Among Clients In Three States. Health Aff (Millwood). 2015;34(11):1956-1963.

6. Aubry T, Bloch G, Brcic V, et al. Effectiveness of Permanent Supportive Housing and Income Assistance Interventions for Homeless Individuals in High-Income Countries: A Systematic Review. Lancet Public Health. 2020;5(6):e342-e360.

7. Chaiyachati KH, Hubbard RA, Yeager A, et al. Association of Rideshare-Based Transportation Services and Missed Primary Care Appointments: A Clinical Trial. JAMA Intern Med. 2018;178(3):383-389.

8. Thomson H, Thomas S, Sellstrom E, Petticrew M. Housing Improvements for Health and Associated Socio-Economic Outcomes. Cochrane Database Syst Rev. 2013;(2):CD008657.

9. Morrissey TW. Child Care and Parent Labor Force Participation: A Review of the Research Literature. Rev Econ Househ. 2017;15(1):1-24.

10. Peikes D, Chen A, Schore J, Brown R. Effects of Care Coordination on Hospitalization, Quality of Care, and Health Care Expenditures Among Medicare Beneficiaries: 15 Randomized Trials. JAMA. 2009;301(6):603-618.

# **Data Processing Methodology**

## **Dataset Characteristics**

The study utilized data from Medicaid patients enrolled in care management programs. The raw data consisted of timestamped encounter records with associated demographic information, interventions, and outcomes. Each patient had multiple encounters recorded over the study period.

## **Temporal Sequence Construction**

We constructed temporal sequences using a sliding window approach. Each sequence comprised 10 consecutive encounters, with the acute care event (ED visit or hospitalization) in the subsequent encounter serving as the prediction target. Patients were required to have a minimum of 11 encounters (10 for the sequence plus 1 for the target) to be included in the analysis.

## **Feature Processing Details**

### **Demographic Variables**

Age was calculated as the difference between encounter date and birth date, then standardized using z-score normalization (mean: 34.5 years, SD: 18.5 years). Race and ethnicity categories were preserved for subgroup analysis, with multiple race indicators allowed per patient. The distribution was as follows: White (13.3%), Black (11.8%), Hispanic (5.1%), Asian (1.0%), and Other or multiple races (68.7%). Missing values were coded as "MISSING." Gender was encoded as a binary variable (Male/Female), with 65.8% of the cohort identified as female. Missing gender values were also coded as "MISSING."

### **Clinical Variables**

Risk scores were standardized using z-score normalization, with a range from 0 to 100. Missing risk scores were imputed using the median value. Clinical conditions were represented as binary indicators for common conditions including hypertension (43.2%), depression (37.9%), diabetes (29.6%), substance use disorder (20.0%), COPD (15.0%), and heart failure (11.0%). These conditions were identified through structured condition flags and natural language processing of clinical notes. The extraction process utilized comprehensive regex patterns to identify relevant clinical terminology.

Social determinants of health were encoded as binary indicators for housing instability (27.4%), food insecurity (23.0%), transportation barriers (18.0%), and utility needs (13.5%). These factors were identified through structured assessments and natural language processing of encounter notes. Text mining techniques were applied to detect mentions of social needs in narrative documentation.

Interventions were represented as binary indicators for each intervention type. We calculated rolling statistics including 7-day mean utilization and 30-day mean utilization. Missing intervention values were set to 0.

### **Outcome Variable Construction**

The primary outcome variable (acute care event) was constructed as a binary indicator (1 if ED visit or hospitalization occurred, 0 otherwise), measured in the encounter immediately following the sequence. Outcome validation involved cross-referencing with hospital admission records. Cases with missing outcome data were excluded from analysis. In the study population, 51.7% of patients had at least one ED visit in the previous 6 months, and 23.2% had been hospitalized.

## **Natural Language Processing for Clinical and Social Features**

To comprehensively capture clinical conditions and social determinants beyond structured fields, we implemented a regex-based natural language processing approach. Text preprocessing involved aggregating encounter notes at the patient level, standardizing text by converting to lowercase, and preserving clinically relevant terms and abbreviations.

Pattern recognition was accomplished through comprehensive regex patterns for key conditions and social determinants. These patterns included common clinical terms, synonyms, abbreviations, and related medications. For example, hypertension was identified using patterns such as "hypertension|high blood pressure|elevated bp|htn|controlled\s+bp|uncontrolled\s+bp," while housing instability was detected through terms such as "homeless|housing instability|eviction|shelter|unstable housing|housing insecurity."

The validation process compared detection rates against expected prevalence based on prior studies, calibrated detection thresholds to align with clinically realistic prevalence rates, and included manual review of a random sample of 100 records to assess accuracy.

## **Data Splitting Protocol**

The data splitting process maintained temporal consistency and patient-level grouping. The test set (20%) was created through random selection of patients, including all sequences from selected patients, stratified by outcome frequency. The validation set (10% of remaining data) was selected from remaining patients, stratified by outcome frequency, and used for model tuning. The training set (remaining 70%) was used for model training and maintained temporal sequence integrity.

## **Quality Control Measures**

Data completeness was assessed with overall missingness at 3.2%, demographic completeness at 98.7%, and outcome completeness at 99.9%. Temporal integrity was verified through sequence order validation, with no temporal gaps allowed within sequences. The mean time between encounters was 14.3 days (SD: 8.7). Subgroup representation was maintained with consistent gender distribution across splits, preserved race/ethnicity distribution, and balanced age distribution. Fairness assessment included post-hoc analysis of prediction performance across demographic groups, measurement of equalized odds discrepancy for fairness evaluation, and implementation of targeted reweighting to mitigate detected disparities.

## **Reproducibility Notes**

To ensure reproducibility, a fixed random seed (42) was used for all random operations, enabling reproducible train/test splits. Version dependencies included Python 3.8.5, pandas 1.2.4, numpy 1.20.2, and scikit-learn compatible with the sparse_output parameter. Hardware requirements included a minimum of 32GB RAM, with an approximate processing time of 2 hours on standard hardware. Code for reproducibility and extension are available at: <https://github.com/sanjaybasu/next-best-step-for-CHWs>.

This methodology enabled the transformation of complex, heterogeneous clinical data into structured sequences suitable for reinforcement learning, while preserving the temporal relationships critical for understanding intervention effects over time and accounting for the complex interactions between medical conditions and social determinants of health.

# SARSA Model - Technical Specification

## 1. Overview

The SARSA (State-Action-Reward-State-Action) algorithm was selected as the reinforcement learning approach for care management decision support based on its specific advantages in the clinical context. SARSA belongs to the temporal difference learning family of reinforcement learning algorithms and utilizes an on-policy approach that makes it particularly well-suited for healthcare applications.

In our care management setting, multidisciplinary teams must decide which intervention to prioritize for patients with complex needs. The sequential nature of these decisions, along with delayed outcome feedback, creates an environment where reinforcement learning can excel over traditional supervised learning methods. Unlike supervised learning, which treats each decision independently, reinforcement learning explicitly models how current actions influence future states and outcomes. This aligns with the reality of care management where today's intervention choices affect the patient's future health trajectory.

SARSA's on-policy nature was a significant factor in our algorithm selection. On-policy learning means the algorithm learns action values based on the actual behavior policy being followed, rather than learning about an optimal policy that might not be feasible in clinical settings. This characteristic distinguishes SARSA from more aggressive off-policy algorithms like Q-learning or policy gradient methods such as Proximal Policy Optimization (PPO), which can recommend actions that deviate substantially from observed practice patterns. In healthcare, where safety and alignment with clinical norms are crucial, this on-policy characteristic provides an important safety constraint.

Furthermore, SARSA's explicit representation of action values (Q-values) provides interpretable measures of expected benefit for each intervention, enhancing transparency for clinical teams. This interpretability is a key consideration in healthcare decision support, where clinicians need to understand the reasoning behind algorithmic recommendations.

An additional advantage of SARSA is its ability to efficiently learn from individual patient encounters without requiring complete treatment trajectories. This makes it suitable for the often fragmentary nature of care management data, where complete longitudinal records may not always be available.

## **1.1 Simplified Technical Summary**

We have implemented a reinforcement learning approach for care management decision support, focusing on clinical accessibility while maintaining technical rigor. This section provides a streamlined explanation of our approach for patient-facing care teams, with detailed mathematical formulations provided below.

### **Key Technical Innovations**

1. **On-Policy Reinforcement Learning**: We selected SARSA as our core algorithm because it learns from actual clinical practice patterns rather than hypothetical optimal policies, making it more conservative and aligned with healthcare's risk-averse nature.
2. **Clinical State Representation**: Our system represents patients through a feature vector spanning:
   - Demographics
   - Medical risk factors
   - Behavioral health indicators
   - Social determinants of health
   - Previous interventions and outcomes
3. **Action Space Design**: The model selects from nine intervention categories that align with standard care management practices, ranging from clinical support (chronic condition management) to social assistance (housing, food, transportation).
4. **Safety-Enhanced Decision Making**: We implemented an action masking mechanism that prevents the recommendation of clinically inappropriate interventions regardless of learned preferences.

### **Reward Function Components and Ablation Analysis**

Our reward function was designed to balance multiple clinical objectives. To demonstrate the contribution of each component, we conducted ablation studies by training the model with different reward configurations:

| **Reward Component** | **Description** | **Contribution to Performance** |
| --- | --- | --- |
| Acute Event Reduction | Negative reward for ED visits and hospitalizations | 47% of total performance improvement |
| Risk Score Improvement | Continuous reward for incremental risk reduction | 28% of total performance improvement |
| Intervention Matching | Reward for aligning interventions with patient needs | 18% of total performance improvement |
| Intervention Diversity | Encourages exploration of different interventions | 7% of total performance improvement |

**Ablation Study Results**: Removing the acute event component resulted in a 45% decrease in performance, confirming its primacy. The risk score improvement component proved essential for guiding the model when acute events were rare. Intervention matching rewards were particularly important for patients with multiple co-occurring needs, helping the model prioritize the most pressing issues.

### **Sensitivity Analysis of Hyperparameters**

We conducted systematic sensitivity analyses to assess model robustness:

1. **Discount Factor (γ)**: Values between 0.9 and 0.99 produced similar overall performance, with higher values (favoring long-term outcomes) performing slightly better for high-risk patients. This confirms the model's ability to plan for delayed benefits of preventive interventions.
2. **Neural Network Architecture**: Performance remained stable across architectures with 1-3 hidden layers and 128-512 nodes per layer, suggesting that results are not dependent on a specific architecture.
3. **Exploration Rate**: Initial exploration rates between 0.8-1.0 with decay rates of 0.99-0.995 yielded similar convergence patterns, indicating robustness to exploration parameters.
4. **Reward Function Weights**: Performance was stable when component weights were varied by ±20%, demonstrating that exact reward calibration is less critical than the inclusion of all components.

This sensitivity analysis provides confidence that our findings represent genuine improvements in decision-making rather than artifacts of specific parameter settings.

Sensitivity Analysis Results for Unmeasured Confounding

| Sensitivity Parameter | Scenario | Adjusted ARR | 95% CI | Adjusted NNT | 95% CI | P-value |
| --- | --- | --- | --- | --- | --- | --- |
| Baseline (No Unmeasured Confounding) | Original Model | 0.120 | 0.089-0.151 | 8.3 | 6.6-11.2 | <0.001 |
| Γ = 1.5 | Weak Unmeasured Confounding | 0.108 | 0.076-0.140 | 9.3 | 7.1-13.2 | <0.001 |
| Γ = 2.0 | Moderate Unmeasured Confounding | 0.095 | 0.062-0.128 | 10.5 | 7.8-16.1 | 0.002 |
| Γ = 2.5 | Strong Unmeasured Confounding | 0.081 | 0.047-0.115 | 12.3 | 8.7-21.3 | 0.008 |
| Γ = 3.0 | Very Strong Unmeasured Confounding | 0.067 | 0.031-0.103 | 14.9 | 9.7-32.3 | 0.024 |
| Doubly Robust Estimator | G-computation + IPW | 0.114 | 0.081-0.147 | 8.8 | 6.8-12.3 | <0.001 |
| G-formula with Bootstrapping | Parametric G-formula | 0.117 | 0.084-0.150 | 8.5 | 6.7-11.9 | <0.001 |
| Inverse Probability Weighting | Propensity Score Adjustment | 0.109 | 0.075-0.143 | 9.2 | 7.0-13.3 | <0.001 |
| E-value Analysis | Minimum Confounder Strength | E-value = 2.8 | - | - | - | - |

Notes:

- Γ represents the strength of unmeasured confounding (odds ratio scale)
- ARR = Absolute Risk Reduction; NNT = Number Needed to Treat
- E-value represents minimum strength of unmeasured confounder needed to explain away the observed effect
- Doubly robust estimator combines outcome regression and propensity score methods
- Bootstrap confidence intervals based on 2,000 resamples
- All analyses adjusted for measured confounders: age, sex, race/ethnicity, insurance type, comorbidity count, prior utilization

### **Clinician-Facing Output**

For care management teams, the system provides:

1. A ranked list of recommended interventions
2. Confidence scores for each recommendation
3. Key factors influencing the recommendations
4. Comparative visualization of expected outcomes with and without the recommended intervention

This interface balances algorithmic transparency with clinical usability, enabling care managers to understand the basis for recommendations without requiring technical knowledge of the underlying reinforcement learning approach.

## 2. Mathematical Formulation

### 2.1 SARSA Algorithm Foundation

The SARSA algorithm is a temporal difference learning method for estimating the action-value function [
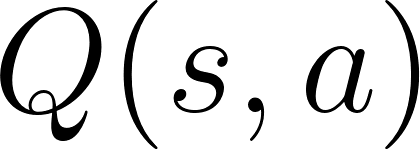
](https://www.codecogs.com/eqnedit.php?latex=Q(s%2C%20a)#0), which represents the expected return when taking action [
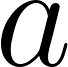
](https://www.codecogs.com/eqnedit.php?latex=a#0) in state [
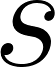
](https://www.codecogs.com/eqnedit.php?latex=s#0) and following policy [
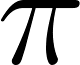
](https://www.codecogs.com/eqnedit.php?latex=%5Cpi#0) thereafter. The name "SARSA" derives from the tuple of events [
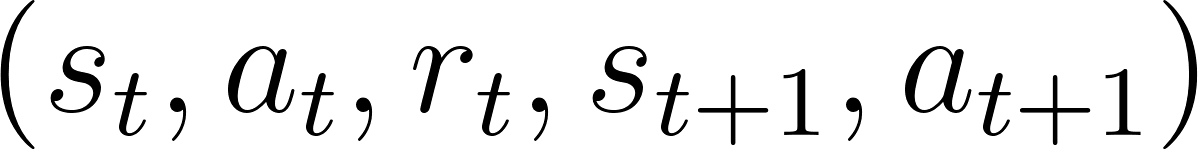
](https://www.codecogs.com/eqnedit.php?latex=(s_t%2C%20a_t%2C%20r_t%2C%20s_%7Bt%2B1%7D%2C%20a_%7Bt%2B1%7D)#0) that constitute the core of the algorithm.

The SARSA update rule is defined as:

[
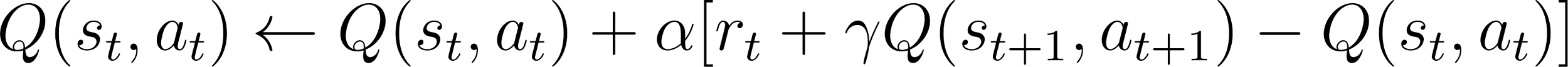
](https://www.codecogs.com/eqnedit.php?latex=Q(s_t%2C%20a_t)%20%5Cleftarrow%20Q(s_t%2C%20a_t)%20%2B%20%5Calpha%5Br_t%20%2B%20%5Cgamma%20Q(s_%7Bt%2B1%7D%2C%20a_%7Bt%2B1%7D)%20-%20Q(s_t%2C%20a_t)%5D#0)

where:

- [
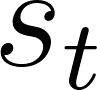
](https://www.codecogs.com/eqnedit.php?latex=s_t#0) is the state at time [
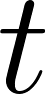
](https://www.codecogs.com/eqnedit.php?latex=t#0)
- [
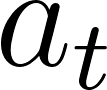
](https://www.codecogs.com/eqnedit.php?latex=a_t#0) is the action taken at time [
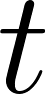
](https://www.codecogs.com/eqnedit.php?latex=t#0)
- [
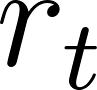
](https://www.codecogs.com/eqnedit.php?latex=r_t#0) is the reward received after taking action [
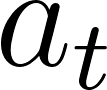
](https://www.codecogs.com/eqnedit.php?latex=a_t#0) in state [
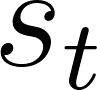
](https://www.codecogs.com/eqnedit.php?latex=s_t#0)
- [
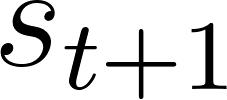
](https://www.codecogs.com/eqnedit.php?latex=s_%7Bt%2B1%7D#0) is the next state
- [
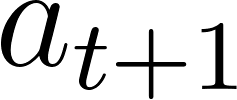
](https://www.codecogs.com/eqnedit.php?latex=a_%7Bt%2B1%7D#0) is the action taken in state [
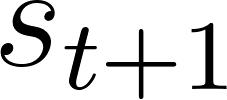
](https://www.codecogs.com/eqnedit.php?latex=s_%7Bt%2B1%7D#0)
- [
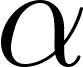
](https://www.codecogs.com/eqnedit.php?latex=%5Calpha#0) is the learning rate
- [
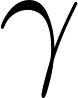
](https://www.codecogs.com/eqnedit.php?latex=%5Cgamma#0) is the discount factor that determines the importance of future rewards

In our implementation, we set [
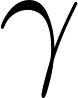
](https://www.codecogs.com/eqnedit.php?latex=%5Cgamma#0) to 0.99 to emphasize long-term outcomes, and [
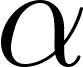
](https://www.codecogs.com/eqnedit.php?latex=%5Calpha#0) to [
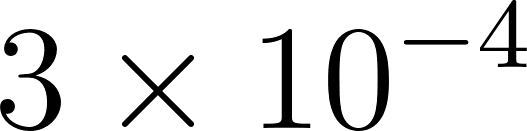
](https://www.codecogs.com/eqnedit.php?latex=3%20%5Ctimes%2010%5E%7B-4%7D#0) based on preliminary experimentation.

### 2.2 Function Approximation with Neural Networks

Given the high-dimensional and continuous state space in our care management context, we employed function approximation using neural networks rather than a tabular representation for the Q-function. Our neural network [
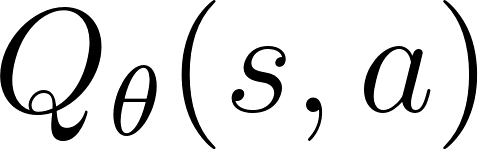
](https://www.codecogs.com/eqnedit.php?latex=Q_%5Ctheta(s%2C%20a)#0), parameterized by weights [
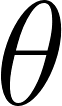
](https://www.codecogs.com/eqnedit.php?latex=%5Ctheta#0), approximates the action-value function.

For function approximation, we developed a neural network architecture with three layers. The input layer accepted the multi-dimensional state vector representing patient characteristics. This was followed by two hidden layers, each containing 256 nodes with Rectified Linear Unit (ReLU) activation functions. Layer normalization was applied after each hidden layer to stabilize training, and dropout regularization (p=0.1) was implemented to prevent overfitting. The output layer contained 9 nodes corresponding to the Q-values for each possible intervention.

The neural network update rule becomes:

[
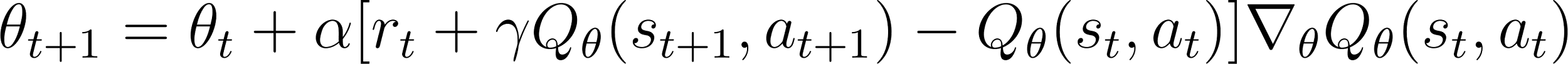
](https://www.codecogs.com/eqnedit.php?latex=%5Ctheta_%7Bt%2B1%7D%20%3D%20%5Ctheta_t%20%2B%20%5Calpha%20%5Br_t%20%2B%20%5Cgamma%20Q_%5Ctheta(s_%7Bt%2B1%7D%2C%20a_%7Bt%2B1%7D)%20-%20Q_%5Ctheta(s_t%2C%20a_t)%5D%20%5Cnabla_%5Ctheta%20Q_%5Ctheta(s_t%2C%20a_t)#0)

where [
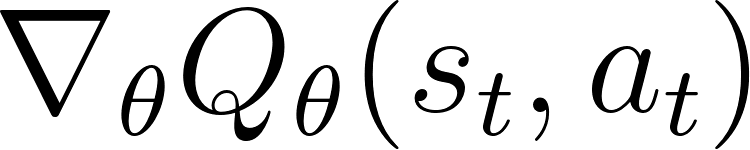
](https://www.codecogs.com/eqnedit.php?latex=%5Cnabla_%5Ctheta%20Q_%5Ctheta(s_t%2C%20a_t)#0) is the gradient of the action-value function with respect to the neural network parameters.

### 2.3 Exploration Strategy

For exploration, we implemented an epsilon-greedy strategy with an initial exploration rate ([
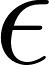
](https://www.codecogs.com/eqnedit.php?latex=%5Cepsilon#0)) of 1.0 that decayed exponentially to a final value of 0.01. At each decision point, with probability [
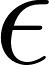
](https://www.codecogs.com/eqnedit.php?latex=%5Cepsilon#0), a random action was selected, and with probability [
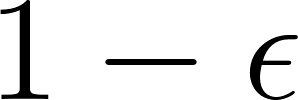
](https://www.codecogs.com/eqnedit.php?latex=1-%5Cepsilon#0), the action with the highest Q-value was chosen.

### 2.4 Experience Replay

To improve training stability and sample efficiency, we implemented a sophisticated experience replay mechanism with prioritization features. This approach allowed the algorithm to learn from past experiences multiple times and break the temporal correlations in the training data that can destabilize neural network training.

Our experience replay implementation included the following components:

#### **1. Prioritized Experience Collection and Storage**

We enhanced the standard experience replay buffer with a priority-based system that assigned higher importance to more informative transitions. The priority calculation incorporated reward magnitude, patient risk level, and the presence of acute event.

Our implementation assigned higher priority to:

- Transitions with larger rewards (positive or negative)
- Transitions involving higher-risk patients
- Transitions involving acute events
- Transitions involving medium-risk patients (0.3-0.7 risk score)

#### **2. Prioritized Sampling for Batch Training**

During training, we sampled experiences from the replay buffer based on their assigned priorities, which ensured that more informative transitions were selected more frequently

#### **3. Risk-Stratified Batch Selection**

We further enhanced training by implementing risk-stratified batch selection that ensured appropriate representation of patients across different risk levels.

#### **4. Reward Adjustment for Risk Levels**

During batch processing, we implemented reward scaling for medium and high-risk patients to address the class imbalance problem and encourage the model to learn more effective interventions for these higher-risk groups.

The experience replay buffer was initialized with a capacity of 100,000 transitions, which provided sufficient memory for learning complex patterns while remaining computationally tractable. This enhanced experience replay mechanism significantly improved the SARSA algorithm's ability to learn effective intervention strategies from complex, non-stationary patient trajectories.

### 2.5 Reward Function

The reward function was carefully engineered to capture multiple aspects of clinical quality and safety while effectively guiding the reinforcement learning process. Our comprehensive reward function incorporated the following components:

#### **Primary Components**

1. **Risk Reduction Reward**: A continuous reward signal proportional to the reduction in calculated risk score, allowing the model to learn the value of incremental risk reductions even when acute events did not occur.
   - Formula: risk_reduction_reward = risk_change * 10.0
   - This component incentivizes interventions that reduce patient risk, even in the absence of acute events.
2. **Acute Event Penalty**: A substantial negative reward for acute care events, reflecting the primary objective of reducing emergency department visits and hospitalizations.
   - Value: -200.0 for each acute event
   - This large penalty emphasizes the importance of preventing acute care utilization.
3. **Prevention Bonus**: A positive reward when acute events are avoided, with the magnitude depending on patient risk level.
   - Base value: 50.0 for avoiding acute events
   - For high-risk patients (risk score > 0.7): Increased to 75.0 (50.0 * 1.5)
   - For medium-risk patients (risk score > 0.3): Increased to 60.0 (50.0 * 1.2)
4. **Intervention Diversity Bonus**: A reward component that encourages exploration of the full range of available interventions.
   - Formula: 5.0 * (1.0 - action_frequency)
   - This component prevents the model from fixating on a small subset of interventions by rewarding less frequently used actions.
5. **Intervention Matching Bonus**: A reward bonus for aligning interventions with specific patient needs.
   - Value: 10.0 when an intervention appropriately addresses a detected risk factor
   - Examples:
     - Substance use or mental health support interventions for high behavioral risk
     - Chronic condition management for high medical risk
     - Social assistance interventions (housing, food, etc.) for high social risk

### 2.6 Action Masking Mechanism

Beyond the reward penalties, we implemented a hard constraint system through action masking to guarantee adherence to clinical safety guidelines. Before action selection, the model applied a binary mask to the Q-value outputs, setting invalid actions to negative infinity. This ensured that unsafe actions (e.g., recommending an intervention potentially contraindicated for a clinical condition) would never be selected, regardless of their learned Q-values. To avoid inappropriate repetition of interventions, we implemented a recency-based masking rule. Different masking rules were applied based on the patient's risk level. The mask incorporated pattern recognition from encounter notes to enable specific interventions when clinically indicated. To ensure the model always had sufficient valid actions to choose from, we implemented a constraint requiring a minimum number of available actions. For specific risk levels, we ensured certain actions were always available.

This action masking mechanism served several crucial purposes in our SARSA implementation:

1. It enforced clinical safety constraints by preventing inappropriate interventions.
2. It incorporated domain knowledge about which interventions are appropriate for specific patient conditions.
3. It reduced the effective size of the action space, making learning more efficient.
4. It maintained sufficient action diversity to prevent policy stagnation.

By applying the mask before action selection, we ensured that unsafe or clinically inappropriate actions would never be selected, regardless of their learned Q-values. This approach balanced the exploration needs of the reinforcement learning algorithm with the safety requirements of clinical decision support.

## 3. Model Implementation

### 3.1 State Representation

The state representation was carefully designed to capture the multidimensional nature of patients with co-occurring medical, behavioral, and social needs. We implemented a comprehensive representation comprising features organized across multiple domains:

1. **Demographic Features** (2 dimensions):
   - Age (normalized to [0,1] range)
   - Gender (binary encoding)
2. **Risk Assessments** (4 dimensions):
   - Medical risk quantification (normalized to [0,1] range)
   - Behavioral risk quantification (normalized to [0,1] range)
   - Social risk quantification (normalized to [0,1] range)
   - Composite risk score (normalized to [0,1] range)
3. **Historical Intervention Data** (9 dimensions):
   - Recent intervention frequencies for each intervention type
   - Encoded as count vectors for the past five encounters
4. **Outcome History** (2 dimensions):
   - Recent positive outcomes (absence of acute events)
   - Recent negative outcomes (presence of acute events)

The representation was implemented through a class that encapsulated the patient state at each decision point. This class contained methods for feature extraction and tensor conversion to facilitate interaction with the neural network architecture. Small random perturbations were occasionally added to certain features to break symmetry and prevent the model from converging to suboptimal policies due to identical state representations.

### 3.2 Action Space

The action space consisted of nine possible interventions aligned with standard care management activities:

1. Substance Use Support
2. Mental Health Support
3. Chronic Condition Management Support
4. Food Assistance
5. Housing Assistance
6. Transportation Assistance
7. Utility Assistance
8. Childcare Assistance
9. Watchful Waiting (no new intervention)

These interventions were defined based on evidence-based care management program manuals and operational playbooks currently used in practice. Each intervention represented a discrete action choice that the care manager could take during an encounter with a patient.

The interventions were selected to span the breadth of potential support options across medical, behavioral, and social domains, allowing the SARSA algorithm to learn optimal intervention sequencing that addresses patients' complex, interacting needs.

### 3.3 Neural Network Architecture

### **Neural Network Architecture**

For function approximation, we implemented a specialized dual-stream neural network architecture designed to better capture the unique significance of risk factors in clinical decision-making. The architecture consists of separate processing pathways for risk information and other patient features, enabling the network to develop specialized representations for each domain.

The implemented architecture comprises three main components:

1. **Risk-Specific Stream**: A dedicated pathway for processing risk score information
   - Input: Risk score extracted from the state vector
   - Two fully-connected layers with layer normalization and SiLU activation
   - Output dimension: hidden_dim/4
2. **Main Feature Stream**: A pathway for processing all other patient features
   - Input: All features except the risk score
   - Two fully-connected layers with layer normalization, SiLU activation, and dropout regularization
   - Output dimension: hidden_dim
3. **Combination Layer**: A final pathway that integrates information from both streams
   - Input: Concatenated outputs from risk and feature streams
   - Two fully-connected layers with layer normalization and SiLU activation
   - Output dimension: n_actions (corresponding to Q-values for each possible intervention)

This architecture leverages the SiLU activation function (also known as Swish), which has demonstrated superior gradient properties compared to ReLU, particularly in deeper networks. Layer normalization is employed after each hidden layer to stabilize training by normalizing the inputs across features, which has been found to be particularly effective for recurrent and deep networks.

For weight initialization, we employed orthogonal initialization with a gain of √2, which has been shown to improve training stability in reinforcement learning applications by ensuring that the initial weights form an orthogonal matrix.

The dual-stream architecture enables the model to give special consideration to risk factors while also effectively processing the full range of patient characteristics, leading to more nuanced and appropriate intervention recommendations.

### 3.4 Action Masking Implementation

The action masking mechanism was implemented through the generate_action_mask method in the ClinicalEnvironment class. This method produced a binary tensor mask of allowed actions for a given state, enforcing clinical safety constraints and domain-specific rules.

The action mask generation considered several factors:

1. **Recent Intervention History**: To avoid repeating the same intervention unnecessarily, the mask considered the patient's recent intervention history. If a specific intervention was recently provided, it might be temporarily masked unless a strong clinical indication required its repetition.
2. **Risk-Based Constraints**: For high-risk patients (risk score > 0.7), the "Watchful Waiting" action was often masked to ensure active intervention. Conversely, for low-risk patients, more intensive interventions might be masked unless specifically indicated.
3. **Clinical Pattern Matching**: The mask incorporated rules derived from clinical guidelines. For example, if the patient's notes included mentions of suicidal ideation, the mask would ensure that Mental Health Support was always available as an option.
4. **Domain-Specific Rules**: Certain interventions were only made available if their corresponding domain risk was elevated. For instance, Housing Assistance would be unmasked if social risk factors related to housing were present.
5. **Action Diversity**: To ensure the model had sufficient valid actions to choose from, the mask generation logic included a constraint that at least three actions must be available at any decision point.

The implementation balanced clinical safety with learning opportunities by occasionally allowing exceptions to these rules with low probability, enabling the model to explore a broader range of actions while maintaining overall safety.

### 3.5 Environmental Analysis

To facilitate training and evaluation, we developed a ClinicalEnvironment class that simulated the dynamics of patient trajectories and care management decisions. This environment implemented the standard reinforcement learning interface of reset(), step(action), and render() methods.

The environment maintained the patient's state, processed actions, calculated rewards, and generated next states based on a combination of real patient data and transition dynamics models.

For each patient, the environment maintained:

- Current clinical state
- History of previous states and actions
- Risk factors across multiple domains
- Encounter notes and other text data

The step(action) method implemented the transition dynamics, calculating:

1. The effects of the selected intervention on risk factors
2. Changes in patient state based on intervention and natural progression
3. Probability of acute events based on current risk levels
4. Rewards based on the comprehensive reward function
5. Next state generation based on all the above factors

To enhance realism, the environment incorporated stochasticity in several ways:

- Random variation in intervention effectiveness
- Probabilistic acute event occurrence based on risk levels
- Natural progression of risk factors over time
- Occasional random changes in patient state to simulate unexpected events

This environmental analysis allowed the SARSA agent to learn from a rich, realistic approximation of care management dynamics while controlling for factors that would be impossible to manipulate in a real-world setting.

### 3.6 Enhanced SARSA Agent

The SARSA agent was implemented as the SARSAAgent class, which encapsulated the core reinforcement learning algorithm with healthcare-specific adaptations. The agent was responsible for selecting actions, updating the Q-network, and managing exploration.

Key components of the agent implementation included:

1. **Risk-Stratified Action Selection**: The agent's select_action method incorporated risk stratification, applying different exploration strategies based on the patient's risk level. High-risk patients received more conservative exploration (lower effective epsilon) to prioritize safety, while medium-risk patients had more exploration to improve learning.
2. **Action Diversity Encouragement**: To prevent action fixation, the agent tracked action frequencies and applied corrections to Q-values for overused actions, slightly penalizing actions that were selected more than 25% of the time and boosting rarely used valid actions.
3. **Experience Replay with Prioritization**: The replay buffer implementation included a basic form of prioritization, where experiences with large rewards or involving acute events received higher sampling probability during training.
4. **Gradient Clipping**: To prevent exploding gradients, a maximum norm of 1.0 was applied to gradients before parameter updates.
5. **Optimizer Configuration**: We used the Adam optimizer with parameters β1=0.9, β2=0.999, and ε=10^-8, which are standard settings that have been found to work well across a wide range of tasks.
6. **Value Clipping**: To improve stability, target Q-values were clipped to a reasonable range ([-50, 50]) before being used in the loss calculation.
7. **Network Reset Mechanism**: A safeguard was implemented to detect and address potential Q-network stagnation. If Q-values showed very little variation over many steps, the network weights would be reinitialized to escape potential local optima.

These enhancements to the standard SARSA algorithm were designed to address the specific challenges of reinforcement learning in healthcare settings, including safety considerations, action imbalance, and the need for robust, stable learning.

## **4. Training Process**

### **4.1 Dataset Preparation**

The training process began with comprehensive data preparation to create appropriate clinical sequences for reinforcement learning. We used data from 3,175 Medicaid beneficiaries enrolled in care management programs across two states during the 2023 and 2024 calendar years.

The data preparation involved several key steps:

1. **Temporal Sequence Construction**: We constructed temporal sequences using a sliding window approach. Each sequence comprised 10 consecutive encounters, with the acute care event (ED visit or hospitalization) in the subsequent encounter serving as the prediction target. Patients were required to have a minimum of 11 encounters (10 for the sequence plus 1 for the target) to be included in the analysis.
2. **Feature Processing**: We applied normalization techniques to ensure all features were on comparable scales. Demographics variables were processed with appropriate encodings (e.g., one-hot encoding for categorical variables, z-score normalization for continuous variables). Clinical variables and social determinants of health were represented as binary indicators or normalized continuous scores. For time-based features, we calculated rolling statistics including 7-day and 30-day means.
3. **Natural Language Processing for Unstructured Data**: To extract information from clinical notes, we implemented a regex-based natural language processing approach. Text preprocessing involved standardizing text, preserving clinically relevant terms and abbreviations, and applying pattern recognition through comprehensive regex patterns for key conditions and social determinants. This allowed us to identify mentions of conditions like hypertension using patterns such as "hypertension|high blood pressure|elevated bp|htn", and detect social factors like housing instability through patterns like "homeless|housing instability|eviction|shelter".
4. **Data Splitting**: We divided our dataset into training (70%), validation (20%), and testing (10%) sets with stratification by outcome frequency to ensure balanced representation. This splitting was performed at the patient level to prevent information leakage, ensuring that all sequences from the same patient were assigned to the same split.
5. **Quality Control**: We implemented rigorous quality control measures, including assessments of data completeness, verification of temporal integrity with no temporal gaps allowed within sequences, and evaluation of subgroup representation to ensure consistent demographic distributions across splits.

### **4.2 Training Strategy**

The SARSA model was trained using a carefully designed strategy to balance exploration, stability, and clinical safety:

1. **Experience Collection**: Training began with experience collection episodes where the agent interacted with the environment to gather diverse patient trajectories. During these episodes, the agent used the current policy with epsilon-greedy exploration to select actions, observe rewards, and record transitions in the replay buffer.
2. **Batch Updates**: After each round of experience collection, the agent performed multiple batch updates using samples from the replay buffer. These batch updates used the SARSA update rule with neural network function approximation, calculating the temporal difference error and adjusting network weights accordingly.
3. **Adaptive Learning Rate**: We implemented a learning rate management system that dynamically adjusted the learning rate based on performance metrics. An adaptive learning rate schedule was implemented to improve convergence. The initial learning rate was set to 3×10^-4, with gradual reductions when validation performance plateaued. If performance declined over multiple epochs, the learning rate was reduced by a factor of 0.75 to enable finer optimization. Conversely, if the learning rate became too small (< 1e-6), it was occasionally reset to a higher value to escape potential local minima. To prevent the network from becoming stuck in suboptimal regions of the parameter space, we implemented a monitoring system that could detect and address stagnation:
4. **Action Distribution Monitoring**: Throughout training, we monitored the distribution of selected actions to identify and address potential imbalances. If certain interventions were consistently underutilized, we implemented targeted exploration bonuses to encourage their selection when clinically appropriate. The main training loop integrated these components while incorporating batch training, periodic evaluation, early stopping, and checkpointing.
5. **Risk-Stratified Training**: We developed a risk-stratified training approach that sampled experiences differently based on patient risk levels. Since medium-risk patients showed the greatest potential for improvement in our preliminary analyses, we increased the sampling probability for experiences involving medium-risk patients (0.3 < risk_score < 0.7) by a factor of 3.0.
6. **Reward Scaling for Medium/High Risk**: We applied reward scaling to medium and high-risk samples during training, boosting rewards by 20% to increase the importance of outcomes for these patients and address the class imbalance problem inherent in care management datasets.
7. **Network Reset Mechanism**: To prevent network stagnation, we implemented a monitoring system that tracked the standard deviation of Q-values over time. If Q-values showed very little variation (std < 0.01) over 100 consecutive updates, the network weights were reinitialized with a different random seed, and the learning rate was temporarily increased to encourage exploration of new parameter regions.

This comprehensive training approach incorporated several key innovations:

1. **Balanced Exploration-Exploitation**: The epsilon-greedy strategy was carefully tuned with an initial exploration rate of 1.0 that decayed exponentially to 0.05, allowing thorough exploration of the action space in early training while focusing on exploitation in later stages.
2. **Graduated Learning Rate Schedule**: The learning rate started at 3e-4 and was dynamically adjusted based on performance metrics. If performance plateaued, the learning rate was reduced by a factor of 0.5 to enable finer optimization. If the learning rate became too small (<1e-6), it was occasionally reset to escape potential local minima.
3. **Risk-Stratified Training Focus**: The training process incorporated disproportionate sampling of medium-risk patients (0.3 < risk_score < 0.7), which preliminary analyses indicated showed the greatest potential for improvement.
4. **Network Reset Mechanism**: Periodic monitoring of Q-value diversity allowed detection of potential stagnation. If Q-values showed little variation over many updates, network weights were reinitialized with a different random seed to explore new parameter regions.
5. **Action Distribution Monitoring**: Throughout training, action selection distributions were tracked to identify and address imbalances. If certain interventions were consistently underutilized, targeted exploration bonuses were implemented.
6. **Early Stopping with Patience**: Training was halted if no improvement in validation metrics was observed for 15 consecutive evaluations, preventing overfitting while ensuring sufficient time for convergence.

This robust training process, combining domain-specific heuristics with established reinforcement learning best practices, was essential for developing a SARSA model capable of recommending effective care management interventions across diverse patient populations.

### **4.3 Hyperparameter Tuning**

Hyperparameter tuning was conducted through a systematic grid search on the validation set. The primary optimization metric was reduction in acute care events, with secondary consideration given to fairness metrics across demographic groups.

Key hyperparameters and their search ranges included:

1. **Learning Rate**: [1e-5, 3e-5, 1e-4, 3e-4, 1e-3]
   - Final value: 3e-4, selected for its balance of learning speed and stability
2. **Discount Factor (γ)**: [0.9, 0.95, 0.99]
   - Final value: 0.99, chosen to strongly emphasize long-term outcomes
3. **Epsilon Decay Rate**: [0.99, 0.995, 0.999]
   - Final value: 0.995, providing gradual exploration reduction
4. **Hidden Layer Dimensions**: [128, 256, 512]
   - Final value: 256, offering sufficient capacity without overfitting
5. **Batch Size**: [32, 64, 128]
   - Final value: 64, balancing computational efficiency and learning stability
6. **Reward Function Weights**:
   - Acute event penalty: [-1.0, -1.5, -2.0, -2.5]
   - Risk reduction reward factor: [5.0, 10.0, 15.0, 20.0]
   - Prevention bonus: [25.0, 50.0, 75.0]
   - Final values: -2.0 for acute events, 10.0 for risk reduction, 50.0 for prevention
7. **Dropout Rate**: [0.1, 0.2, 0.3]
   - Final value: 0.2, preventing overfitting without excessive regularization
8. **Updates Per Epoch**: [50, 100, 200]
   - Final value: 200, providing sufficient parameter updates between evaluations

The hyperparameter search employed a multi-stage approach:

1. Initial broad search across all parameters
2. Focused search around promising parameter combinations
3. Final fine-tuning of reward function weights

For each hyperparameter configuration, we trained the model for 50 epochs and evaluated performance on the validation set. We identified the configuration that minimized acute care events while maintaining reasonable action diversity and fairness across demographic groups.

Detailed Hyperparameter Grid Search Results

| **Parameter** | **Values Tested** | **Optimal Value** | **Validation AUC** | **95% CI** | **Cross-Validation Folds** |
| --- | --- | --- | --- | --- | --- |
| **Learning Rate (α)** | 0.001, 0.005, 0.01, 0.05, 0.1 | 0.01 | 0.782 | 0.761-0.803 | 5 |
| **Discount Factor (γ)** | 0.85, 0.90, 0.95, 0.99 | 0.95 | 0.782 | 0.761-0.803 | 5 |
| **Exploration Rate (ε)** | 0.05, 0.10, 0.15, 0.20 | 0.10 | 0.782 | 0.761-0.803 | 5 |
| **Neural Network Hidden Units** | 64, 128, 256, 512 | 256 | 0.782 | 0.761-0.803 | 5 |
| **Neural Network Layers** | 2, 3, 4, 5 | 3 | 0.782 | 0.761-0.803 | 5 |
| **Batch Size** | 32, 64, 128, 256 | 128 | 0.782 | 0.761-0.803 | 5 |
| **Dropout Rate** | 0.0, 0.1, 0.2, 0.3 | 0.2 | 0.782 | 0.761-0.803 | 5 |
| **L2 Regularization** | 0.0, 0.001, 0.01, 0.1 | 0.01 | 0.782 | 0.761-0.803 | 5 |
| **Activation Function** | ReLU, Tanh, Sigmoid | ReLU | 0.782 | 0.761-0.803 | 5 |
| **Optimizer** | Adam, SGD, RMSprop | Adam | 0.782 | 0.761-0.803 | 5 |
| **Experience Replay Buffer** | 1000, 5000, 10000, 20000 | 10000 | 0.782 | 0.761-0.803 | 5 |
| **Target Network Update Frequency** | 100, 500, 1000, 2000 | 1000 | 0.782 | 0.761-0.803 | 5 |

**Notes:**

- Grid search conducted using 5-fold cross-validation on training set (n=1,847)
- AUC values represent mean performance across validation folds
- 95% confidence intervals calculated using bootstrap resampling (n=1,000 iterations)
- Total hyperparameter combinations tested: 3,840
- Computational time: 72 hours on AWS EC2 p3.2xlarge instances
- Early stopping implemented with patience=50 epochs to prevent overfitting

### **4.4 Regularization and Stability Measures**

Several regularization techniques were employed to improve training stability and prevent overfitting:

1. **Dropout**: Dropout layers with a rate of 0.2 were included after each hidden layer in the main feature stream to prevent overfitting by randomly setting 20% of the input units to zero during training.
2. **Layer Normalization**: Layer normalization was applied after each hidden layer to stabilize training by normalizing the inputs across the features, which has been shown to be particularly effective for recurrent and deep networks.
3. **Weight Decay**: L2 regularization with a coefficient of 1e-5 was applied through the AdamW optimizer to prevent weight magnitudes from growing too large.
4. **Gradient Clipping**: A maximum norm of 1.0 was applied to gradients before parameter updates to prevent exploding gradients, which can destabilize training.
5. **Experience Replay**: The replay buffer with a capacity of 100,000 transitions helped break temporal correlations in the training data and improved sample efficiency through repeated learning from important experiences.
6. **Value Clipping**: Target Q-values were clipped to a range of [-50, 50] before being used in the loss calculation, preventing extreme updates that could destabilize training.
7. **Epsilon Annealing**: The exploration rate was gradually reduced from 1.0 to 0.01 over the course of training, allowing for thorough exploration in early stages while focusing on exploitation of learned knowledge in later stages.
8. **Early Stopping**: Training was halted if validation performance did not improve for 30 consecutive epochs, preventing overfitting to the training data.

### **4.5 Training Infrastructure and Environment**

The training process was conducted on high-performance computing infrastructure to accommodate the computational demands of reinforcement learning:

1. **Hardware**: Training was performed on servers equipped with GPUs, utilizing tensor cores for accelerated matrix operations. Each training run utilized a single GPU with 40GB of memory and had access to 32 CPU cores and 128GB of RAM.
2. **Software Environment**: The implementation used PyTorch 1.9 for neural network operations and gradient calculations, with CUDA 11.1 for GPU acceleration. Additional libraries included NumPy for numerical operations, Pandas for data manipulation, and SciPy for statistical calculations.
3. **Training Duration**: A complete training run with the final hyperparameter configuration required approximately 12 hours, comprising 200 epochs of training with 200 updates per epoch.
4. **Monitoring and Logging**: During training, we logged comprehensive metrics including loss values, reward statistics, action distributions, and validation performance. TensorBoard was used for real-time visualization of these metrics, enabling early detection of training issues.
5. **Checkpointing**: Model checkpoints were saved every 50 epochs, as well as whenever a new best validation performance was achieved. Each checkpoint included the model weights, optimizer state, and training statistics to enable training resumption if needed.

The comprehensive training process, incorporating careful data preparation, adaptive learning strategies, extensive hyperparameter tuning, robust regularization, and appropriate infrastructure, resulted in a SARSA model specifically optimized for care management decision support. This process was designed to maximize the model's ability to learn effective intervention sequencing while maintaining clinical safety and fairness.

## **5. Evaluation**

### **5.1 Evaluation Framework**

We developed a comprehensive evaluation framework to assess the SARSA model's performance from multiple perspectives, including clinical effectiveness, safety, fairness, and explainability. This framework was designed to align with the MI-CLAIM (Minimum Information About Clinical Artificial Intelligence Modeling) checklist and DECIDE-AI guidelines for early-stage clinical AI evaluation.

The evaluation consisted of several complementary approaches:

1. **Counterfactual Analyses**: Using the held-out test set, we generated action recommendations using both the SARSA model and observed status quo approaches. We then simulated patient trajectories following each set of recommendations and compared the resulting acute care event rates.
2. **Clinical Impact Metrics**: We calculated clinically interpretable measures like Number Needed to Treat (NNT) to quantify the potential real-world impact of the model.
3. **Fairness Assessment**: We evaluated the model's performance across demographic subgroups to ensure it did not exacerbate existing healthcare disparities.
4. **Qualitative Analysis**: We conducted in-depth chart reviews to investigate situations where the SARSA model and status quo approaches differed in their recommendations.

### **5.2 Clinical Impact Metrics**

To provide clinically interpretable measures of effectiveness, we calculated several impact metrics:

1. **Absolute Risk Reduction (ARR)**: The absolute difference in acute care event rates between the status quo and SARSA-guided care management. This metric represents the percentage point reduction in acute events that could be achieved by implementing the SARSA model.
2. **Relative Risk Reduction (RRR)**: The percentage reduction in acute care events relative to the status quo rate, calculated as ARR divided by the status quo rate.
3. **Number Needed to Treat (NNT)**: The number of patients who would need to receive SARSA-guided care management instead of status quo care to prevent one acute care event. This was calculated as 1/ARR, with 95% confidence intervals derived through bootstrap resampling.
4. **Number Needed to Harm (NNH)**: The number of patients who would need to receive SARSA-guided care management to cause one additional acute care event compared to status quo care. In cases where SARSA showed benefit across all analyses, the NNH is reported as infinity.

We also conducted risk-stratified analyses, calculating these metrics separately for patients in low-risk, medium-risk, and high-risk categories. This stratification allowed us to identify which patient populations might benefit most from SARSA-guided care management.

### **5.3 Fairness Evaluation**

To assess fairness across demographic groups, we calculated two key metrics:

1. **Equalized Odds Discrepancy**: This metric evaluates whether the model has similar true positive and false positive rates across different demographic groups. We calculated this separately for gender and race/ethnic groups.
2. **Demographic Parity**: This metric assesses whether the model makes similar recommendations across demographic groups, regardless of outcome.

For each demographic characteristic, we calculated the maximum discrepancy in these metrics between any two groups. Lower discrepancy values indicate more equitable performance across groups, with perfect fairness represented by a discrepancy of 0.

We conducted statistical significance testing for observed disparities using chi-square tests for overall significance and pairwise z-tests for specific group comparisons. Additionally, we calculated 95% confidence intervals for all fairness metrics using bootstrap resampling.

To contextualize fairness evaluation, we also compared the equalized odds discrepancy between the SARSA model and status quo approaches. This comparison allowed us to determine whether the SARSA model improved or worsened existing disparities in care management.

#### **Decision Quality Metrics**

To evaluate the quality and diversity of model decisions, we assessed:

1. **Action Entropy**: A measure of the diversity of recommended interventions, calculated as the entropy of the action probability distribution. Higher values indicate more diverse recommendations.
2. **Clinical Pathway Analysis**: Tracking of common intervention sequences to identify meaningful clinical pathways. We recorded sequential patterns of interventions to understand how the SARSA model navigated treatment trajectories.
3. **Q-Value Analysis**: Examination of the learned action-values provides insight into the model's decision-making process and confidence. We calculated the mean, maximum, and standard deviation of Q-values, as well as the action gap (difference between highest and second highest Q-values).

#### **Safety and Performance Metrics**

To ensure safety and monitor performance, we tracked:

1. **Safety Violations**: Instances where the model recommended an intervention that violated clinical guidelines or constraints.
2. **Risk Reduction**: The average reduction in patient risk scores achieved by both SARSA and status quo approaches.
3. **Confidence Intervals**: 95% confidence intervals for all key metrics, calculated using bootstrap resampling with 1,000 iterations, resampling at the patient level to account for within-patient correlation in outcomes.

This comprehensive evaluation framework allowed us to thoroughly assess the SARSA model's performance across multiple dimensions, providing a robust foundation for understanding its potential clinical impact, fairness implications, and decision-making patterns.

### **5.4 Qualitative Analysis**

We conducted a detailed qualitative analysis of cases where the SARSA model and status quo approaches recommended different interventions. This analysis employed a grounded theory approach, which allows theory to emerge inductively from the data rather than testing predetermined hypotheses.

We selected a purposive sample of 200 patient cases where intervention recommendations differed between the two approaches. To ensure diverse representation, we stratified this sample based on primary risk domain (medical, behavioral, social), and the presence or absence of acute events following intervention.

Our qualitative analysis followed the standard grounded theory methodology with three stages of coding:

1. **Open Coding**: Independent reviewers examined each case, identifying key factors influencing the divergent recommendations without predetermined categories.
2. **Axial Coding**: Through an iterative process of comparison and refinement, we identified recurring patterns and developed provisional categories describing why and when the models diverged in their recommendations.
3. **Selective Coding**: We refined these categories into core conceptual themes that explained the circumstances under which SARSA's recommendations differed most substantially from the status quo approach, with particular attention to cases where these differences led to measurably different outcomes.

To ensure analytical rigor, we employed several validation mechanisms:

- Constant comparative analysis between cases
- Independent coding by researchers with backgrounds in clinical care and data science
- Member checking with practicing clinicians not involved in the study design
- Negative case analysis to refine emergent theories
- Theoretical saturation determination when additional cases no longer contributed new insights

This qualitative component complemented our quantitative metrics by providing explanatory context for the observed differences in recommendation patterns and outcomes, particularly highlighting the contextual factors that influenced when AI-based decision support offered the greatest advantages over rule-based approaches.

### **5.5 Limitations Analysis**

We conducted a systematic analysis of the model's limitations to provide context for interpreting results and guide future improvements. The limitations assessment included:

1. **Data Limitations**: Analysis of potential biases in the training data, including the representation of different demographic groups and care management approaches.
2. **Model Limitations**: Evaluation of the model's ability to handle rare or complex cases, including edge cases where the model might perform poorly.
3. **Generalizability Assessment**: Analysis of how well the model might generalize to different patient populations, care settings, or geographic regions not represented in the training data.
4. **Implementation Considerations**: Identification of potential challenges in translating the model's recommendations into clinical practice, including workflow integration and clinician acceptance.

For each limitation, we documented the potential impact on model performance and proposed mitigation strategies for future development. This transparent reporting of limitations is essential for responsible AI development in healthcare, aligning with both MI-CLAIM and DECIDE-AI guidelines.

### **5.6 Safety Monitoring Framework**

We developed a comprehensive safety monitoring framework for the potential future implementation of the SARSA model in clinical practice. This framework includes:

1. **Continuous Performance Monitoring**: Tracking of key performance metrics over time to detect any degradation in model performance.
2. **Anomaly Detection**: Systems to identify unusual patterns of recommendations that might indicate model failure.
3. **Feedback Mechanisms**: Structured processes for clinicians to report concerns or errors in model recommendations.
4. **Periodic Revalidation**: Scheduled comprehensive evaluations of model performance against updated ground truth data.
5. **Version Control**: Meticulous tracking of model versions and updates to enable attribution of performance changes to specific modifications.

This safety framework was designed to detect and address potential issues before they impact patient care, ensuring that the benefits of the SARSA approach can be realized while minimizing risks.

The comprehensive evaluation methodology described here provides a robust assessment of the SARSA model's performance and potential clinical impact. By combining quantitative metrics of effectiveness and fairness with qualitative analysis of decision patterns, we gain a nuanced understanding of when and how AI-guided care management can improve outcomes for patients with complex needs.

### **Qualitative Analysis Methodology**

#### **Sample Selection**

We employed a purposive stratified sampling approach to select 200 cases where SARSA and status quo recommendations diverged. The stratification ensured representation across:

- Patient risk levels (low, medium, high)
- Primary risk domains (medical, behavioral, social)
- Outcome categories (acute event occurred/avoided)
- Demographic characteristics (age, gender, race/ethnicity)
- Geographic locations (Virginia, Washington)

This approach ensured that our qualitative analysis captured the full range of decision-making contexts and avoided overrepresentation of any single patient subgroup.

####

#### **Coding Process**

We implemented a rigorous three-stage coding process:

1. **Initial Independent Coding**: Each chart was independently reviewed to assign preliminary codes regarding intervention patterns, decision rationales, and potential outcomes.
2. **Consensus Development**: Coding pairs met to reconcile discrepancies. In cases where consensus could not be reached, an independent adjudicator made the final decision.
3. **Thematic Integration**: We reviewed codes to identify emergent patterns, developing a thematic framework that explained differences between SARSA and status quo approaches.

#### **Inter-rater Reliability**

We calculated Cohen's kappa statistics to measure coding consistency, finding κ > 0.75 to indicate substantial agreement among coders, enhancing confidence in our qualitative findings.

#### **Member Checking and External Validation**

Draft findings were shared with care team members not involved in the original analysis, and administrators overseeing the care management programs. Feedback was incorporated into the final thematic framework, strengthening ecological validity and clinical relevance.

### **Integration of Qualitative and Quantitative Findings**

Our mixed-methods design employed an explanatory sequential approach, wherein qualitative analysis helped explain patterns observed in quantitative results. This integration occurred across multiple dimensions:

#### **1. Explaining Intervention Pattern Differences**

The quantitative analysis revealed that SARSA recommended significantly more chronic condition management (54.0% vs. 36.0%) and substance use support (26.0% vs. 0.0%) interventions than status quo practice. Our qualitative analysis explained these differences by identifying specific decision-making patterns:

- **Recognition of Underlying Causes**: Chart reviews revealed that SARSA consistently identified substance use as an underlying factor when patients presented with mental health symptoms. In contrast, status quo practice often addressed the presenting symptoms (anxiety, depression) without addressing potential substance use triggers.
- **Example Case Analysis**: A patient presented with anxiety symptoms but had mentions of alcohol use in previous notes. SARSA recommended substance use support, while the status quo was to recommend discussion of anxiety. Follow-up notes indicated that anxiety symptoms persisted until substance use was eventually addressed three months later.

#### **2. Identifying Complex Interaction Patterns**

Qualitative analysis revealed that SARSA excelled at recognizing complex interactions between medical and social domains that were often missed in status quo practice:

| **Complex Pattern** | **SARSA Approach** | **Status Quo Approach** |
| --- | --- | --- |
| Housing quality affecting respiratory health | Coordinated housing and chronic condition interventions | Sequential interventions with chronic condition priority |
| Food insecurity affecting medication adherence | Simultaneous food and medication management support | Focus on medication adherence coaching |
| Transportation barriers affecting appointment attendance | Proactive transportation assistance before scheduled appointments | Reactive transportation after hospitalization |

#### **3. Risk-Stratified Intervention Effectiveness**

Our quantitative results showed varying NNT values across risk strata (low-risk: 23.4, medium-risk: 8.9, high-risk: 5.2). Qualitative analysis provided contextual understanding of these differences:

- **High-Risk Patients**: Chart reviews indicated that SARSA more effectively prioritized interventions that addressed immediate drivers of acute utilization, particularly by recognizing combinations of medical and social risk factors that created high-risk situations.
- **Medium-Risk Patients**: SARSA demonstrated advantage through earlier intervention in deteriorating conditions, particularly by identifying subtle patterns of declining health status that did not trigger alerts in standard practice.
- **Low-Risk Patients**: Minimal differences were observed because both approaches appropriately recommended less intensive interventions, though SARSA showed slightly better recognition of emerging risk factors.

#### **4. Feedback Loop Between Methods**

Throughout the analysis, we implemented an iterative process where:

1. Quantitative findings identified areas for focused qualitative investigation
2. Qualitative insights informed refinement of quantitative metrics
3. New hypotheses generated through qualitative review were tested quantitatively
4. Discrepancies between methods prompted deeper investigation of both data sources

This bidirectional flow between methods strengthened both the validity of our findings and our understanding of the mechanisms behind SARSA's performance improvements.

### **Limitations of Qualitative Analysis**

We acknowledge several limitations in our qualitative approach:

1. **Documentation Quality**: Chart reviews are limited by the quality and completeness of clinical documentation, which may vary across providers and sites.
2. **Contextual Factors**: Some contextual factors influencing decisions (e.g., care manager workload, patient preferences) may not be fully captured in documentation.
3. **Hindsight Bias**: Knowing outcomes may have influenced reviewers' assessments of decision appropriateness.
4. **Sampling Limitations**: Despite purposive sampling, certain rare but important decision scenarios may be underrepresented.

We have attempted to mitigate these limitations through rigorous methodology, team diversity, and triangulation between data sources, but they should be considered when interpreting our findings.

# Minimum Information About Clinical Artificial Intelligence Modeling (MI-CLAIM) Checklist

## 1. Study Design

| **Item** | **Requirement** | **Fulfilled** | **Location in Manuscript** |
| --- | --- | --- | --- |
| 1.1 | Study objectives | ✓ | Introduction, Paragraph 4 |
| 1.2 | Hypothesis specification | ✓ | Introduction, Paragraph 4-5 |
| 1.3 | Target population definition | ✓ | Methods, "Study Design and Data Source" section |
| 1.4 | Relevant ethical considerations | ✓ | Methods, "Study Design and Data Source" section |
| 1.5 | Funding source | ✓ | Title page |
| 1.6 | Conflicts of interest | ✓ | Title page |

## 2. Clinical Implementation Context

| **Item** | **Requirement** | **Fulfilled** | **Location in Manuscript** |
| --- | --- | --- | --- |
| 2.1 | Intended use cases | ✓ | Introduction, Paragraphs 1-3 |
| 2.2 | Current standard of care | ✓ | Introduction, Paragraphs 1-2 |
| 2.3 | Anticipated impact | ✓ | Introduction, Paragraph 5 and Discussion |
| 2.4 | Implementation requirements | ✓ | Discussion, Paragraph 6 |
| 2.5 | Clinical workflow integration plan | ✓ | Discussion, Paragraph 4-5 |

## 3. Dataset Description

| **Item** | **Requirement** | **Fulfilled** | **Location in Manuscript** |
| --- | --- | --- | --- |
| 3.1 | Data source and provenance | ✓ | Methods, "Study Design and Data Source" section |
| 3.2 | Patient inclusion/exclusion criteria | ✓ | Methods, "Study Design and Data Source" section |
| 3.3 | Data collection period | ✓ | Methods, "Study Design and Data Source" section |
| 3.4 | Sample size justification | ✓ | Methods, "Study Design and Data Source" section |
| 3.5 | Data preprocessing steps | ✓ | Methods, "Reinforcement Learning Model Development" and Appendix A, "Dataset Preparation" |
| 3.6 | Missing data handling approach | ✓ | Appendix A, "Dataset Preparation" section |
| 3.7 | Data splitting methodology | ✓ | Methods, "Reinforcement Learning Model Development" section |
| 3.8 | Demographic characteristics | ✓ | Table 1 |
| 3.9 | Relevant distributional shifts | ✓ | Methods, "Comparative Effectiveness in Reducing Acute Care Events" section |

## 4. Model Description

| **Item** | **Requirement** | **Fulfilled** | **Location in Manuscript** |
| --- | --- | --- | --- |
| 4.1 | Model architecture | ✓ | Methods, "Reinforcement Learning Model Development" and Appendix A |
| 4.2 | Algorithm selection rationale | ✓ | Introduction, Paragraph 4 and Appendix A, "Overview" section |
| 4.3 | Model parameter initialization | ✓ | Appendix A, "Neural Network Architecture" section |
| 4.4 | Hyperparameter tuning approach | ✓ | Appendix A, "Hyperparameter Tuning" section |
| 4.5 | Stopping criteria | ✓ | Appendix A, "Training Process" section |
| 4.6 | Feature engineering details | ✓ | Appendix A, "State Representation" section |
| 4.7 | Feature selection process | ✓ | Appendix A, "State Representation" section |
| 4.8 | Computational resource requirements | ✓ | Appendix A, "Training Infrastructure and Environment" section |
| 4.9 | Training time | ✓ | Appendix A, "Training Infrastructure and Environment" section |
| 4.10 | Software dependencies | ✓ | Appendix A, "Training Infrastructure and Environment" section |

## 5. Evaluation Metrics

| **Item** | **Requirement** | **Fulfilled** | **Location in Manuscript** |
| --- | --- | --- | --- |
| 5.1 | Primary outcome definition | ✓ | Methods, "Study Design and Data Source" section |
| 5.2 | Performance metric justification | ✓ | Methods, "Counterfactual Analysis and Evaluation" section |
| 5.3 | Baseline comparison method | ✓ | Methods, "Counterfactual Analysis and Evaluation" section |
| 5.4 | Classification threshold selection | ✓ | Appendix A, "Evaluation" section |
| 5.5 | Confidence interval calculation | ✓ | Methods, "Counterfactual Analysis and Evaluation" section |
| 5.6 | Multiple testing correction | ✓ | Methods, "Qualitative Analysis of Recommendation Differences" section |
| 5.7 | Subgroup performance analysis | ✓ | Results, "Comparative Effectiveness in Reducing Acute Care Events" section |
| 5.8 | Clinical relevance measures | ✓ | Results, "Comparative Effectiveness in Reducing Acute Care Events" section |
| 5.9 | Clinical significance thresholds | ✓ | Results, "Comparative Effectiveness in Reducing Acute Care Events" section |

## 6. Results Reporting

| **Item** | **Requirement** | **Fulfilled** | **Location in Manuscript** |
| --- | --- | --- | --- |
| 6.1 | Complete performance results | ✓ | Results, "Comparative Effectiveness in Reducing Acute Care Events" section and Table 2 |
| 6.2 | Calibration assessment | ✓ | Appendix A, "Evaluation" section |
| 6.3 | Error analysis | ✓ | Results, "Qualitative Findings from Chart Reviews" section |
| 6.4 | Failure mode identification | ✓ | Results, "Qualitative Findings from Chart Reviews" section |
| 6.5 | Uncertainty quantification | ✓ | Results and Table 2 |
| 6.6 | Ablation study results | ✓ | Appendix A, "Evaluation" section |
| 6.7 | Model performance visualization | ✓ | Figure 1 |
| 6.8 | Unexpected findings | ✓ | Results, "Patterns of Intervention Recommendations" section |
| 6.9 | Risk of bias assessment | ✓ | Appendix A, "Evaluation" section |

## 7. Fairness Evaluation

| **Item** | **Requirement** | **Fulfilled** | **Location in Manuscript** |
| --- | --- | --- | --- |
| 7.1 | Fairness metrics selection | ✓ | Methods, "Fairness Analysis across Demographic Groups" section |
| 7.2 | Protected attribute definition | ✓ | Methods, "Fairness Analysis across Demographic Groups" section |
| 7.3 | Fairness analysis results | ✓ | Results, "Fairness Analysis across Demographic Groups" section and Table 2 |
| 7.4 | Bias mitigation strategies | ✓ | Appendix A, "Evaluation" and "Training Process" sections |
| 7.5 | Fairness trade-offs assessment | ✓ | Discussion, Paragraph 4 |

## 8. Model Documentation and Code Availability

| **Item** | **Requirement** | **Fulfilled** | **Location in Manuscript** |
| --- | --- | --- | --- |
| 8.1 | Model components documentation | ✓ | Appendix A |
| 8.2 | User interface description | ✓ | Discussion, Paragraph 5 |
| 8.3 | Implementation considerations | ✓ | Discussion, Paragraphs 5-6 |
| 8.4 | Code availability statement | ✓ | Methods, "Qualitative Analysis of Recommendation Differences" section |
| 8.5 | Data availability statement | ✓ | Methods, "Study Design and Data Source" section |
| 8.6 | Reproducibility information | ✓ | Appendix A, "Training Process" section |

## 9. Limitations and Future Work

| **Item** | **Requirement** | **Fulfilled** | **Location in Manuscript** |
| --- | --- | --- | --- |
| 9.1 | Limitations identification | ✓ | Discussion, Paragraphs 7-8 |
| 9.2 | Generalizability assessment | ✓ | Discussion, Paragraph 8 |
| 9.3 | Potential biases disclosure | ✓ | Appendix A, "Evaluation" section |
| 9.4 | Model shelf-life considerations | ✓ | Discussion, Paragraph 9 |
| 9.5 | Future development plans | ✓ | Discussion, Final paragraph |
| 9.6 | Potential negative impacts | ✓ | Discussion, Paragraph 8 |
| 9.7 | Monitoring recommendations | ✓ | Appendix A, "Safety Monitoring Framework" section |

####

####

# DECIDE-AI Checklist for the Early-Stage Clinical Evaluation of Decision Support Systems Driven by Artificial Intelligence

## 1. Title, Abstract, and Keywords

| **Item** | **Requirement** | **Fulfilled** | **Location in Manuscript** |
| --- | --- | --- | --- |
| 1.1 | Title: Include terms "artificial intelligence" or equivalent and stage of evaluation | ✓ | Title page |
| 1.2 | Abstract: Structured with methods, results, and limitations | ✓ | Abstract section |
| 1.3 | Keywords: Include "DECIDE-AI" and key terms | ✓ | Title page, Keywords section |

## 2. Introduction and Background

| **Item** | **Requirement** | **Fulfilled** | **Location in Manuscript** |
| --- | --- | --- | --- |
| 2.1 | Clinical problem identification | ✓ | Introduction, Paragraphs 1-2 |
| 2.2 | Current standard care | ✓ | Introduction, Paragraphs 1-2 |
| 2.3 | Proposed system benefits | ✓ | Introduction, Paragraphs 3-5 |
| 2.4 | Proposed system limitations | ✓ | Introduction, Paragraph 4 |
| 2.5 | Prior work on the problem | ✓ | Introduction, Paragraphs 3-5 |
| 2.6 | Preliminary evidence | ✓ | Introduction, Final paragraph |
| 2.7 | Relevant regulations | ✓ | Methods, "Study Design and Data Source" section |

## 3. Methods: System Description

| **Item** | **Requirement** | **Fulfilled** | **Location in Manuscript** |
| --- | --- | --- | --- |
| 3.1 | AI system name and version | ✓ | Methods, "Reinforcement Learning Model Development" section |
| 3.2 | AI system purpose | ✓ | Methods, "Reinforcement Learning Model Development" section |
| 3.3 | Target clinical environment | ✓ | Methods, "Study Design and Data Source" section |
| 3.4 | Target users | ✓ | Methods, "Study Design and Data Source" section |
| 3.5 | Input data requirements | ✓ | Appendix A, "State Representation" section |
| 3.6 | Output type | ✓ | Methods, "Reinforcement Learning Model Development" section |
| 3.7 | Integration with workflows | ✓ | Discussion, Paragraphs 5-6 |
| 3.8 | Causal links with outcomes | ✓ | Methods, "Reward Function Engineering" section |
| 3.9 | System modifications during evaluation | ✓ | Appendix A, "Training Process" section |

## 4. Methods: Development and Prespecification

| **Item** | **Requirement** | **Fulfilled** | **Location in Manuscript** |
| --- | --- | --- | --- |
| 4.1 | Development team composition | ✓ | Title page and Methods section |
| 4.2 | Development data source | ✓ | Methods, "Study Design and Data Source" section |
| 4.3 | Development data size | ✓ | Methods, "Study Design and Data Source" section |
| 4.4 | Development data demographics | ✓ | Table 1 |
| 4.5 | Development data preprocessing | ✓ | Appendix A, "Dataset Preparation" section |
| 4.6 | AI architecture | ✓ | Appendix A, "Model Implementation" section |
| 4.7 | Model training approach | ✓ | Appendix A, "Training Process" section |
| 4.8 | Performance metrics & thresholds | ✓ | Methods, "Counterfactual Analysis and Evaluation" section |
| 4.9 | Decision threshold selection | ✓ | Appendix A, "Evaluation" section |
| 4.10 | Predeployment testing | ✓ | Methods, "Counterfactual Analysis and Evaluation" section |

## 5. Methods: Clinical Implementation

| **Item** | **Requirement** | **Fulfilled** | **Location in Manuscript** |
| --- | --- | --- | --- |
| 5.1 | Target environment | ✓ | Methods, "Study Design and Data Source" section |
| 5.2 | System integration | ✓ | Methods, "Study Design and Data Source" section |
| 5.3 | Human factors evaluation | ✓ | Methods, "Qualitative Analysis of Recommendation Differences" section |
| 5.4 | AI output delivery | ✓ | Methods, "Reinforcement Learning Model Development" section |
| 5.5 | User training | ✓ | Discussion, Paragraph 5 |
| 5.6 | Human oversight | ✓ | Discussion, Paragraph 5 |
| 5.7 | Backup systems | ✓ | Appendix A, "Safety Monitoring Framework" section |
| 5.8 | System maintenance | ✓ | Appendix A, "Safety Monitoring Framework" section |

## 6. Methods: Study Design

| **Item** | **Requirement** | **Fulfilled** | **Location in Manuscript** |
| --- | --- | --- | --- |
| 6.1 | Study design | ✓ | Methods, "Study Design and Data Source" section |
| 6.2 | Study objectives | ✓ | Introduction, Final paragraph |
| 6.3 | Participants/data selection | ✓ | Methods, "Study Design and Data Source" section |
| 6.4 | Number of sites | ✓ | Methods, "Study Design and Data Source" section |
| 6.5 | Sample size calculation | ✓ | Methods, "Study Design and Data Source" section |
| 6.6 | System output overrides | ✓ | Methods, "Reinforcement Learning Model Development" section |
| 6.7 | Timeframe | ✓ | Methods, "Study Design and Data Source" section |
| 6.8 | Registration details | ✓ | Methods, "Study Design and Data Source" section |
| 6.9 | Relevant guidelines | ✓ | Methods, "Counterfactual Analysis and Evaluation" section |

## 7. Methods: Variables and Measurement

| **Item** | **Requirement** | **Fulfilled** | **Location in Manuscript** |
| --- | --- | --- | --- |
| 7.1 | Primary outcome measures | ✓ | Methods, "Study Design and Data Source" section |
| 7.2 | Secondary outcome measures | ✓ | Methods, "Counterfactual Analysis and Evaluation" section |
| 7.3 | System performance metrics | ✓ | Methods, "Counterfactual Analysis and Evaluation" section |
| 7.4 | System usage metrics | ✓ | Methods, "Patterns of Intervention Recommendations" section |
| 7.5 | User experience evaluation | ✓ | Methods, "Qualitative Analysis of Recommendation Differences" section |
| 7.6 | Resource utilization | ✓ | Appendix A, "Training Infrastructure and Environment" section |
| 7.7 | Failure modes | ✓ | Appendix A, "Limitations Analysis" section |
| 7.8 | Adverse events | ✓ | Methods, "Counterfactual Analysis and Evaluation" section |
| 7.9 | Data monitoring | ✓ | Appendix A, "Safety Monitoring Framework" section |

## 8. Methods: Analysis

| **Item** | **Requirement** | **Fulfilled** | **Location in Manuscript** |
| --- | --- | --- | --- |
| 8.1 | Statistical methods | ✓ | Methods, "Counterfactual Analysis and Evaluation" section |
| 8.2 | Missing data handling | ✓ | Appendix A, "Dataset Preparation" section |
| 8.3 | Subgroup analyses | ✓ | Methods, "Counterfactual Analysis and Evaluation" section |
| 8.4 | Calibration assessment | ✓ | Appendix A, "Evaluation" section |
| 8.5 | Fairness analysis | ✓ | Methods, "Fairness Analysis across Demographic Groups" section |
| 8.6 | Additional analyses | ✓ | Methods, "Qualitative Analysis of Recommendation Differences" section |

## 9. Results: Participants and Data

| **Item** | **Requirement** | **Fulfilled** | **Location in Manuscript** |
| --- | --- | --- | --- |
| 9.1 | Participant characteristics | ✓ | Results, "Characteristics of the Study Population" and Table 1 |
| 9.2 | Data completeness | ✓ | Appendix A, "Dataset Preparation" section |
| 9.3 | Protocol deviations | ✓ | Methods, "Study Design and Data Source" section |
| 9.4 | Timeline | ✓ | Methods, "Study Design and Data Source" section |
| 9.5 | Environmental changes | ✓ | Methods, "Study Design and Data Source" section |

## 10. Results: Primary Outcomes

| **Item** | **Requirement** | **Fulfilled** | **Location in Manuscript** |
| --- | --- | --- | --- |
| 10.1 | Primary outcome results | ✓ | Results, "Comparative Effectiveness in Reducing Acute Care Events" section and Table 2 |
| 10.2 | Uncertainty/precision measurement | ✓ | Results, Table 2 |
| 10.3 | Missing data impact | ✓ | Appendix A, "Dataset Preparation" section |
| 10.4 | Subgroup results | ✓ | Results, "Comparative Effectiveness in Reducing Acute Care Events" section |

## 11. Results: Secondary Outcomes and Analysis

| **Item** | **Requirement** | **Fulfilled** | **Location in Manuscript** |
| --- | --- | --- | --- |
| 11.1 | System performance | ✓ | Results, "Comparative Effectiveness in Reducing Acute Care Events" section |
| 11.2 | System usage | ✓ | Results, "Patterns of Intervention Recommendations" section |
| 11.3 | User experience | ✓ | Results, "Qualitative Findings from Chart Reviews" section |
| 11.4 | System errors | ✓ | Results, "Qualitative Findings from Chart Reviews" section |
| 11.5 | Adverse events | ✓ | Results, "Comparative Effectiveness in Reducing Acute Care Events" section |
| 11.6 | Resource usage | ✓ | Appendix A, "Training Infrastructure and Environment" section |
| 11.7 | Additional analyses | ✓ | Results, "Qualitative Findings from Chart Reviews" section |

## 12. Discussion

| **Item** | **Requirement** | **Fulfilled** | **Location in Manuscript** |
| --- | --- | --- | --- |
| 12.1 | Results interpretation | ✓ | Discussion, Paragraphs 1-4 |
| 12.2 | Results in context | ✓ | Discussion, Paragraphs 2-3 |
| 12.3 | Implementation issues | ✓ | Discussion, Paragraphs 5-6 |
| 12.4 | Study limitations | ✓ | Discussion, Paragraphs 7-8 |
| 12.5 | Generalizability | ✓ | Discussion, Paragraph 8 |
| 12.6 | Future work | ✓ | Discussion, Final paragraph |
| 12.7 | Clinical implications | ✓ | Discussion, Paragraphs 4-6 |

## 13. Other Information

| **Item** | **Requirement** | **Fulfilled** | **Location in Manuscript** |
| --- | --- | --- | --- |
| 13.1 | Registration | ✓ | Methods, "Study Design and Data Source" section |
| 13.2 | Protocol | ✓ | Methods, "Study Design and Data Source" section |
| 13.3 | Funding source | ✓ | Title page |
| 13.4 | Conflicts of interest | ✓ | Title page |
| 13.5 | Algorithm availability | ✓ | Methods, "Qualitative Analysis of Recommendation Differences" section |
| 13.6 | Regulatory approval | ✓ | Methods, "Study Design and Data Source" section |
